# Microglia in Alzheimer’s disease: pathogenesis, mechanisms, and therapeutic potentials

**DOI:** 10.3389/fnagi.2023.1201982

**Published:** 2023-06-15

**Authors:** Jifei Miao, Haixia Ma, Yang Yang, Yuanpin Liao, Cui Lin, Juanxia Zheng, Muli Yu, Jiao Lan

**Affiliations:** ^1^Shenzhen Bao’an Traditional Chinese Medicine Hospital, Shenzhen, China; ^2^School of Chemical Biology and Biotechnology, Peking University Shenzhen Graduate School, Shenzhen, China; ^3^Shenzhen Bao’an Traditional Chinese Medicine Hospital, Guangzhou University of Chinese Medicine, Shenzhen, China

**Keywords:** Alzheimer’s disease, microglia, amyloid-beta, phagocytosis, neuroinflammation

## Abstract

Alzheimer’s disease (AD) is a neurodegenerative disorder characterized by protein aggregation in the brain. Recent studies have revealed the critical role of microglia in AD pathogenesis. This review provides a comprehensive summary of the current understanding of microglial involvement in AD, focusing on genetic determinants, phenotypic state, phagocytic capacity, neuroinflammatory response, and impact on synaptic plasticity and neuronal regulation. Furthermore, recent developments in drug discovery targeting microglia in AD are reviewed, highlighting potential avenues for therapeutic intervention. This review emphasizes the essential role of microglia in AD and provides insights into potential treatments.

## 1. Introduction

Alzheimer’s disease (AD) is a progressive neurodegenerative disorder characterized by cognitive decline, memory loss, and changes in behavior and mood. The accumulation of Aβ and tau proteins in the brain is thought to play a significant role in AD pathogenesis. Microglia plays a multifaceted role in AD, contributing to inflammation, phagocytosis, and neurodegeneration. In response to Aβ plaque accumulation, microglia become activated and produce proinflammatory cytokines, leading to a chronic state of neuroinflammation (Hansen et al., 2018). This inflammation has been shown to worsen neurodegeneration and contribute to the progression of AD (Leng and Edison, 2021). Furthermore, as AD progresses, microglia’s ability to phagocytose Aβ plaques decreases, leading to their accumulation in the brain and further AD progression (Hansen et al., 2018). Additionally, microglia contribute to neurodegeneration in AD through the release of toxic substances and regulation of synaptic function (Hong et al., 2016). Understanding the role of microglia in AD is crucial for developing effective treatments. This review provides a comprehensive summary of microglial involvement in AD, including the relevant mutated genes, microglial subtypes, phagocytic function, inflammatory response, and neurodegeneration. It also discusses recent developments in therapy development targeting microglia in AD. Further research is necessary to fully understand the mechanisms by which microglia contribute to AD and to develop new strategies for the treatment of this debilitating disease.

## 2. Mutant genes in microglia associated with AD

Emerging evidence suggests that mutations in genes associated with microglia significantly impact the development of AD. Microglia play a critical role in monitoring for signs of damage or infection in the brain and responding appropriately. However, mutations in microglia-associated genes can lead to altered microglial functions, including cytokine secretion and phagocytic activity, resulting in increased inflammation in the brain and contributing to AD progression. Moreover, microglial dysfunction has been linked to the clearance of Aβ peptides, key contributors to AD pathology. Mutations in microglia-associated genes may impair this clearance process, leading to the accumulation of Aβ peptides and further cognitive decline. Furthermore, changes in gene expression and intracellular signaling pathways have been linked to mutations in microglia-associated genes, further impairing microglial function and contributing to AD progression. The relationship between mutations in microglia-associated genes and AD underscores the critical role that microglia play in maintaining brain health and protecting against damage and disease. To fully understand the mechanisms of AD onset and progression, it is essential to continue researching these mutated genes and their effects on microglial function. This review provides a comprehensive overview of the current understanding of the relationship between mutations in microglia-associated genes and AD, highlighting the importance of microglial function in the maintenance of brain health and the potential for microglial-targeted therapies.

### 2.1. *APOE*

The *APOE* gene has three common alleles, ε2, ε3, and ε4, that result from single-nucleotide polymorphisms (Sims et al., 2020). The ε4 allele is a strong risk factor for late-onset Alzheimer’s Disease (LOAD), with individuals carrying one *APOE* ε4 allele having a 3–4-fold higher risk for AD, while those with two ε4 alleles have a 10–15-fold higher risk (Corder et al., 1993; Saunders et al., 1993; Sims et al., 2020). This allele is highly enriched in AD patients and is expressed in both the brain and liver, playing a role in lipoprotein transportation and modulating the inflammatory response (Goldstein and Brown, 2015; Flowers and Rebeck, 2020). The contribution of *APOE4* to AD pathogenesis has been attributed primarily to its induction of early and abundant amyloid pathology. However, recent evidence suggests that *APOE4* can also independently worsen tau pathology and neurodegeneration (Yamazaki et al., 2019; Koutsodendris et al., 2022). Clinical studies have found a correlation between *APOE* ε4 and more severe tau pathology, neurodegeneration, and memory impairment in AD patients (Filippini et al., 2009; Therriault et al., 2020; Wang Y.-T. T. et al., 2021; Weigand et al., 2021). *APOE* ε4 allele is strongly associated with Aβ pathology in human AD brains, with homozygous carriers having the highest Aβ burden (Polvikoski et al., 1995; Premkumar et al., 1996; Bertram et al., 2007; Reiman et al., 2009; Mishra et al., 2018). Reducing APOE levels has been shown to be an attractive strategy for alleviating Aβ pathology in AD patients carrying the *APOE* ε4 allele. Studies using human iPSC-derived neurons and cerebral organoids have shown that *APOE4* exacerbates tau phosphorylation and synapse loss, leading to neurodegeneration (Lin et al., 2018; Wang C. et al., 2018; Zhao et al., 2020a). *APOE4* knock-in mice exhibit loss of hilar GABAergic interneurons and memory deficits (Andrews-Zwilling et al., 2010). Depletion of astrocytic *APOE4* reduces tau pathology and neurodegeneration in *PS19-APOE4* knock-in mice, indicating that reducing *APOE4* levels may be an effective strategy for alleviating tau pathology and neurodegeneration in AD therapy (Wang C. et al., 2021). As a potential therapeutic target, various approaches to reducing *APOE4* are being explored, including converting it to *APOE2* or *APOE3* (Safieh et al., 2019; Yamazaki et al., 2019; Williams et al., 2020; McDade et al., 2021). It is noteworthy that the increase in *APOE* expression in microglia may be protective or detrimental to the disease, and further investigation is needed to determine the stage of the disease when *APOE* reduction may be effective. This review provides a comprehensive overview of the current understanding of the role of *APOE* ε4 in AD, highlighting the potential of reducing *APOE4* levels as a therapeutic strategy for AD.

### 2.2. TREM2

TREM2 is a transmembrane receptor that is predominantly expressed in tissue macrophages, including microglia in the brain. It belongs to the immunoglobulin superfamily and is composed of an extracellular IgV domain, a stalk, a single transmembrane helix, and a cytosolic tail (Kober et al., 2016). TREM2 binds to several ligands, including apoptotic cells, phospholipids, glycolipids, lipidated particles, and lipoproteins (Song et al., 2017). Mutations in *TREM2* are linked to Nasu-Hakola disease (Paloneva et al., 2002), and heterozygous variants have been associated with an increased risk of developing LOAD (Guerreiro et al., 2013; Jonsson et al., 2013). In AD, TREM2 plays a crucial role in regulating microglial activation. Activated microglia form a barrier that limits the spreading and toxicity of Aβ, and increasing TREM2 expression through overexpression of human TREM2 has been shown to reduce Aβ deposition (Lee et al., 2018). Moreover, agonistic antibodies against TREM2 have been shown to reduce Aβ load and improve behavioral performance (Schlepckow et al., 2020; Ellwanger et al., 2021). TREM2 also helps microglia clear myelin debris and promote remyelination, making it a potential therapeutic target for demyelinating diseases (Plemel et al., 2018; Claes et al., 2021; Zirngibl et al., 2022). The soluble form of TREM2, sTREM2, has been found to be a useful marker of AD pathology and cognitive decline, with higher levels correlating positively with neurodegenerative markers and slower cognitive decline (Henjum et al., 2016; Heslegrave et al., 2016; Suárez-Calvet et al., 2016b; Ewers et al., 2019, 2020; Pascoal et al., 2021). However, the impact of TREM2 cleavage and sTREM2 on AD is not yet clear, as some studies have found a protective effect while others have found a detrimental effect. sTREM2 has been found to bind to Aβ aggregates and impact Aβ pathology, and may activate an independent signaling pathway, presumably by activating the extracellular signal-regulated kinase 1/2 (ERK1/2) pathway (Wu et al., 2015; Zhong et al., 2017, 2019). Investigating the role of TREM2 in microglial cells is crucial to better understand the molecular mechanisms underlying AD and to develop new therapies that target microglia and TREM2.

### 2.3. CD33

CD33 is predominantly expressed by microglia in the brain and has been investigated for its association with AD susceptibility. Through genome-wide association studies (GWAS), the rs3826656 single nucleotide polymorphism (SNP) and nearby SNP, rs3865444, have been identified as susceptibility factors for LOAD (Hollingworth et al., 2011; Karch et al., 2012). Post-mortem analyses have shown increased expression of CD33 on microglia in AD brains, which correlates with Aβ burden and cognitive decline (Karch et al., 2012; Griciuc et al., 2013). However, higher CD33 expression in AD could also be a response to disease pathology, such as inflammation (Nomura et al., 2001; Ramsborg and Papoutsakis, 2007). Initially, the rs3865444 SNP, located in the *CD33* promoter, was thought to modulate *CD33* gene expression (Malik et al., 2013). However, further analysis revealed a co-inherited SNP, rs12459419, located in exon 2 of *CD33*, which impacts mRNA splicing and mediates the expression of two isoforms of CD33: a longer isoform, hCD33M, and a shorter isoform, hCD33m. The rs12459419C allele, co-inherited with the rs3865444C allele, results in hCD33M:hCD33m transcript ratios of 9:1, while the AD-protective rs12459419T allele (i.e., rs3865444A) shifts this ratio to 7:3 (Malik et al., 2013). Cells expressing the rs12459419T allele show decreased levels of hCD33M at the protein level (Bradshaw et al., 2013; Malik et al., 2013). Higher levels of hCD33M expression are associated with decreased phagocytosis in various cells, as demonstrated in primary monocytes and cultured mouse microglia BV2 cells (Bradshaw et al., 2013; Griciuc et al., 2013). Recent studies have shown that hCD33M can decrease the uptake of various cargos, including polystyrene beads, dextran, myelin, and aggregated Aβ1-42 (Bhattacherjee et al., 2019, 2021a). Recent studies have reported a gain-of-function role for hCD33m in microglia, leading to increased phagocytosis, cell migration, and cell proliferation while decreasing cell adhesion (Bhattacherjee et al., 2021b). The two hCD33 isoforms appear to play distinct roles in microglia. Another *CD33* SNP, rs2455069, has been proposed to be associated with AD susceptibility, with *in silico* analysis suggesting that an amino acid switch at position 69 of hCD33 from an arginine to glycine in the rare rs2455069 SNP may enhance the affinity for sialic acid-containing ligands (Tortora et al., 2022). However, further testing is needed to establish this association within a larger cohort.

The investigation of CD33’s association with AD susceptibility and its effects on microglia provides insights into the role of microglia in AD pathology. However, the exact mechanisms by which CD33 isoforms influence microglial function and their impact on AD progression remain to be fully elucidated. Further research is necessary to fully understand the role of CD33 in AD and to develop potential therapeutic strategies targeting microglia and CD33 in AD treatment.

### 2.4. Other genes

Early research on AD employed cost-effective approaches such as targeted sequencing to identify rare variants in known AD-related genes. In contrast, more recent studies have dedicated substantial resources to whole exome sequencing (WES) or whole genome sequencing (WGS) to uncover rare variants in previously unidentified loci. The AD Sequencing Project (ADSP) conducted a WGS family-based study and a WES case-control study on over 30,000 samples (Beecham et al., 2017), uncovering rare *SORL1* loss-of-function variants, TREM2^*R*47*H*^, a common variant in PILRA, and a novel rare variant in long non-coding RNA AC099552.4 (Raghavan et al., 2018; Bis et al., 2020). Gene-level analyses implicated *OPRL1* and *GAS2L2* in AD, suggesting transcriptional regulation by ZNF655 (Bis et al., 2020). Another investigation identified a novel variant in the NSF gene associated with AD (Fan et al., 2020), while a weighted burden analysis of ADSP’s WES subjects implicated rare variants in *TREM2*, *ABCA7*, *SORL1*, and *PSEN1* in AD (Curtis et al., 2020). The PI3K/AKT signaling pathway was found to be significantly associated with *TREM2*, with *PIK3R1*, *WNT7A*, *CR1*, and *EXOC5* likely containing detrimental rare variants and *TIAF1* and *NDRG2* potentially harboring protective rare variants (Curtis et al., 2020). The largest exome analysis to date corroborated established rare variants in *TREM2*, *SORL1*, and *ABCA7* loci and identified novel rare variants in the microglial gene ATP8B4 (Holstege, 2020). WGS detected rare variants in non-coding regions, with *ABCA1*, *TMEM132A*, and *AKAP9* segregating with AD in families and *AKAP9* being nominally associated with LOAD risk (Beecham et al., 2018; Vardarajan et al., 2018). Rare variants in *CR1*, *BIN1*, *FERMT2*, and *SLC24A4* were also pinpointed, aligning with genome-wide association study (GWAS) findings (Beecham et al., 2018; Vardarajan et al., 2018). A family-based WGS association study identified *FNBP1L*, *SEL1L*, *LINC00298*, *PRKCH*, *C15ORF41*, *C2CD3*, *KIF2A*, *APC*, *LHX9*, *NALCN*, *CTNNA2*, *SYTL3*, and *CLSTN2*, implicating genes involved in neuroplasticity, synaptic function, and neurodevelopmental pathways, thus broadening the range of AD-related biological pathways (Prokopenko et al., 2021). Collectively, these studies have confirmed and offered novel insights into AD-related biological pathways, emphasizing the significance of WGS in identifying genetic variants in non-coding regions and previously overlooked AD pathogenesis pathways. In this review, we have summarized 37 microglial mutant genes associated with AD (Table 1). Further independent replication of these findings is necessary due to the rarity of some variants.

**TABLE 1:** Genes in microglia associated with AD.

Gene	Variant	Protein description	Related functions
*APOE* (Serrano-Pozo et al., 2021)	3 common variants or isoforms: *APOE2* decreases, although *APOE4* increases LOAD risk relative to *APOE3*	Major apolipoprotein in brain HDL-like particles	Chaperone Aβ and lipids; endocytosis; phagocytosis
*NME8* (Lambert et al., 2013)	rs2718058	Encodes protein with thioredoxin and nucleoside diphosphate kinase domains	Unknown function
*CD33* (Griciuc et al., 2019)	rs12459419	Binds sialylated ligands; phosphorylated ITIM recruits phosphatase SHP-1	ITIM signaling; phagocytosis inhibition
*MEF2C* (Lynch et al., 2005)	rs190982	Widely studied in muscle cells and neurons	Microglia activation
*TREM2* (Liu et al., 2020; Hardy and Salih, 2021)	rare coding LOF variants: R47H, R62H, T66M, H157Y	Binds anionic/lipophilic ligands; triggers DAP12 ITAM to recruit kinase Syk	ITAM signaling; Phagocytosis; inflammation
*INPP5D* (Tsai et al., 2021; Lin et al., 2022)	rs35349669	SH3-containing inositol phosphatase, converts phosphatidylinositol (3,4,5)-trisphosphate to phosphatidylinositol (3,4)-bisphosphate	Endocytosis; phagocytosis
*PLCG2* (Sims et al., 2017; Tsai et al., 2022)	rare P522R coding GOF variant	Phospholipase activity cleaves phosphatidylinositol (4,5)-bisphosphate into IP3 and DAG second messengers	TREM2 signaling; phagocytosis; inflammation
*CD2AP* (Tao et al., 2019)	rs10948363, rs9296559 and rs9349407	Scafolding protein co-localizing with F-actin	Phagocytosis; phagosome formation
*LILRB4* (Salih et al., 2019)	rs731170	ITIM-containing transmembrane receptor	ITIM signaling
*CR1* (Zhu et al., 2015)	rs6656401	Complement receptor 1; binds C1q and C3b/C4b	Complement system cascade; phagocytosis
*EPHA1* (Talebi et al., 2020; Buhl et al., 2022)	rs11771145, rs11767557	Receptor tyrosine kinase for ephrin-A class ligands	Eph/ephrin signaling
*SCIMP* (Schwartzentruber et al., 2021)	rs61182333	Non-TIR-containing TLR adaptor protein	TLR signaling; phagocytosis
*SHARPIN* (Schwartzentruber et al., 2021)	rs78541244	Subunit of LUBAC regulating NF-κB and MAPK signaling	NF-κB pathway; inflammation
*ITGAM* (Salih et al., 2019)	rs79113991	Encode integrin CD11b	Phagocytosis
*PTK2B* (Combs et al., 1999; Li et al., 2016)	rs28834970	Non–receptor tyrosine kinase; homolog of focal adhesion kinase	Actin cytoskeletal rearrangement; phagocytosis
*CASS4* (Beck et al., 2014)	rs7274581	Scaffold protein associated with focal adhesion kinases FAK, Pyk2	Focal adhesion; cytoskeletal rearrangement
*ABI3* (Sekino et al., 2015)	rare coding variant S209F	Component of the Abi/WAVE complex involved in actin polymerization	Motility; phagocytosis
*RIN3* (Kajiho et al., 2011)	rs10498633	a guanine nucleotide exchange factor for Rab5 and Rab31	early endocytic pathway
*PICALM* (Ando et al., 2022)	rs3851179, rs541458	Endocytic adaptor	Endo-lysosomal trafficking; phagocytosis
*FERMT2* (Eysert et al., 2021)	rs17125944	Adaptor between membrane and actin cytoskeleton at extracellular matrix adhesion sites	Actin polymerization; focal adhesion
*BIN1* (Gao et al., 2021)	rs6733839, rs744373	Involved in membrane curvature and dynamin interaction	Endocytosis; phagocytosis
*HLA-DRB1* (Ghosh et al., 2013)	rs9271192	Major histocompatibility complex class II protein for extracellular antigen presentation	Phagocytosis
*RAB10* (Tavana et al., 2019)	rs95684712	Rab superfamily member	Endosomal trafficking and fusion
*SPI1* (Cao et al., 2022)	rs1057233	Transcription factor PU.1	Gene expression
*SORL1* (Mishra et al., 2022)	17 coding variants identified, some common and rare	Receptor for vesicular sorting of lipoproteins and various receptors	Endo-lysosomal network; lipid metabolism;
*SPPL2A* (Lambert et al., 2013; Schwartzentruber et al., 2021)	rs12592778	Aspartyl protease in late endosomes and lysosomes	Inflammation
*GRN* (Schwartzentruber et al., 2021)	rs5848	Lysosomal protein	Lysosomal degradation
*ABCA7* (Zhao et al., 2015)	rs3764650, rare coding LOF variants	ATP-binding cassette transporter; multipass transmembrane protein transports lipids	Phagocytosis
*MS4A* (Deming et al., 2019)	rs983392	Four-pass transmembrane protein in MS4A family	Microglial receptor complex
*ZCWPW1* (Karch et al., 2016)	rs1476679	Presumed epigenetic regulator through its chromatin-binding domains	Immune receptors

## 3. Microglia activation

**FIGURE 1:**
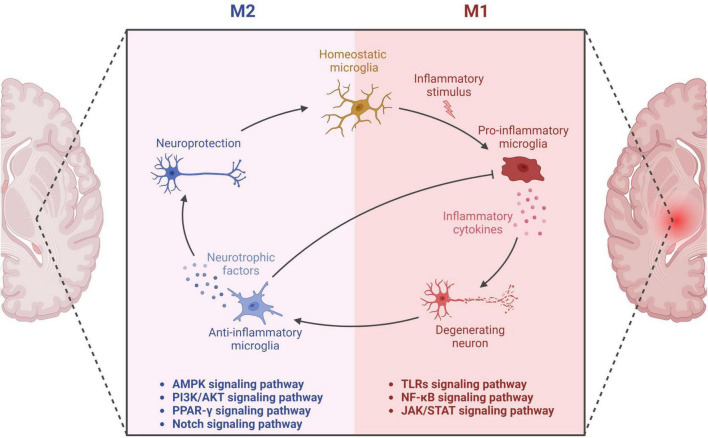
Microglial activation in neurodegeneration and neuroprotection. The diagram showcases the dual functions of microglia through M1 and M2 phenotypes. The M1 microglial phenotype promotes neuroinflammation through the secretion of pro-inflammatory factors, leading to neuronal damage. The related pathways involved in this process encompass Toll-like Receptors (TLRs), Nuclear Factor-kappa B (NF-κB), and the Janus Kinase/Signal Transducers and Activators of Transcription (JAK/STAT) pathway. On the other hand, the M2 phenotype of microglia secretes anti-inflammatory factors, thereby providing neuroprotective effects. This aspect is mediated through various signaling pathways including Adenosine Monophosphate-Activated Protein Kinase (AMPK), Phosphatidylinositol 3-Kinase/Protein Kinase B (PI3K/AKT), Peroxisome Proliferator-Activated Receptor Gamma (PPAR-γ), and the Notch pathway. Thus, the diagram delineates the dual role of microglia in neuronal health and disease, underlining the critical balance between the M1 and M2 states.

Microglia exhibit diverse phenotypes in response to environmental cues and display heterogeneity in their activation, which can be broadly classified into two categories: classical (M1) and alternative (M2) (Sica and Mantovani, 2012; Tang and Le, 2016). M1 activation provokes inflammation and neurotoxicity, while M2 activation elicits anti-inflammatory and reparative responses (Tang and Le, 2016; Colonna and Butovsky, 2017). Microglia can adapt their phenotype to different environments, ensuring a protective role (Du et al., 2018). However, recent transcriptomic investigations reveal that microglial activation is multifaceted, with various intermediate phenotypes between M1 and M2 (Correale, 2014). Researchers have also identified a novel subpopulation of microglia associated with neurodegenerative diseases, termed disease-associated microglia (DAM), which localize around Aβ plaques in AD and exhibit a distinct gene expression profile (Keren-Shaul et al., 2017). DAM activation comprises a two-step process involving TREM2-independent and TREM2-dependent mechanisms (Keren-Shaul et al., 2017). The binary classification of microglia as M1 or M2 has been contested, and researchers propose that a continuum of different intermediate phenotypes more accurately reflects microglial activation states. M2 microglia uptake and remove Aβ deposits, protect against AD, and mitigate neurotoxic Aβ aggregation (Song and Suk, 2017). However, uncontrolled microglial activation and aberrant responses to Aβ can harm neurons, resulting in neuroinflammation, synapse loss, and tau pathology (Tang and Le, 2016; Sarlus and Heneka, 2017; Hansen et al., 2018). Microglia continuously transform among phenotypes, and two peaks of microglial activation in AD have been proposed: an early anti-inflammatory peak during the pre-clinical stage and a later pro-inflammatory peak during the clinical stage as the disease advances (Fan et al., 2017). In summary, proper microglial function is crucial in AD pathology, and dysregulation can have detrimental consequences on neurons. Comprehending the dynamic nature of microglial activation and their dual role in AD pathogenesis is vital for developing effective therapies for AD. In this context, further investigation into the signaling pathways involved in microglial activation, such as TLRs, NF-κB, MAPK, PI3K/AKT, and JAK/STAT pathways, is essential (Figure 1). Understanding these pathways’ roles in microglial function can facilitate the development of targeted therapies for AD.

### 3.1. Pro-inflammatory (M1) polarization

#### 3.1.1. TLRs signaling pathway

The transmembrane pattern-recognition receptor family, including TLR2 and TLR4, exhibits significant expression in microglia (Cui et al., 2020). TLR4 is noted for its ability to bind lipopolysaccharide (LPS), an interaction that sparks pro-inflammatory signaling pathways and M1 microglial polarization, both of which have been implicated in AD’s neuroinflammatory pathogenesis (Yang Z. et al., 2019). This LPS-TLR4 complex initiates a cascade of inflammatory signaling, beginning with TLR4’s association with myeloid differentiation factor 88 (MyD88) and leading to the autophosphorylation of interleukin-1 receptor-associated kinase (IRAK) (Subhramanyam et al., 2019). This subsequently activated IRAK1 and IRAK4 interact with tumor necrosis factor receptor-associated factor-6 (TRAF6) (Subhramanyam et al., 2019), which then activates the transforming growth factor-β-activated kinase-1 (TAK1) complex. This intricate signaling leads to the activation of two major pro-inflammatory pathways, the NF-κB and MAPK, resulting in the transcription of pro-inflammatory genes that contribute to AD pathogenesis (Kawai and Akira, 2007; Subhramanyam et al., 2019). Similarly, TLR2 also plays a critical role in the innate immune system, activating MyD88-mediated signaling (Cui et al., 2020). Interestingly, TLR2-mediated autophagy has been shown to regulate microglial M1/M2 phenotype switching, a process that could potentially modulate the inflammatory responses in AD (Ma et al., 2020). Autophagy can be stimulated by TLR2 signaling, suggesting a potential mechanism for controlling microglial phenotype and, thus, the neuroinflammatory response in AD. In summary, while the LPS-TLR4 complex instigates pro-inflammatory signaling pathways linked to AD, TLR2 signaling might modulate microglial phenotypes and AD’s neuroinflammatory pathogenesis through autophagy. These findings provide valuable insights into the mechanisms of microglial activation in AD and highlight potential therapeutic targets for this devastating neurodegenerative disease.

#### 3.1.2. NF-κB signaling pathway

NF-κB is a pivotal transcription factor in the regulation of microglial activation, a process significantly involved in the inflammatory response seen in AD. The NF-κB p65/p50 subunits, in particular, play a major role in promoting M1 microglial activation. Conversely, inhibiting these subunits can suppress the transcription of inflammatory genes and promote a shift in microglia polarization toward the M2 phenotype (Zhang et al., 2013). This has been substantiated by the identification of numerous modulators that can induce a M1 to M2 phenotype shift in microglia by inhibiting NF-κB, further emphasizing the crucial role this pathway plays in microglial polarization and, by extension, in the inflammatory component of AD pathogenesis (Kawai and Akira, 2007). In the absence of stimulation, NF-κB remains inactive in the cytoplasm by interacting with inhibitors of NF-κB (IκB) proteins (Kawai and Akira, 2007). However, when stimulated by the TAK1 complex, the IκB kinase (IKK) complex catalyzes the phosphorylation of IκB proteins. Phosphorylated IκBs are then polyubiquitinated and degraded, which allows NF-κB p50/p65 to translocate into the nucleus (Kawai and Akira, 2007). There, they bind to target promoters and promote the transcription of pro-inflammatory genes, a process that has been implicated in the chronic neuroinflammation seen in AD (Mulero et al., 2019). In summary, NF-κB plays a crucial role in microglial polarization and the regulation of inflammatory gene transcription, both of which are significant in the context of AD. Greater understanding of the pathways influencing NF-κB activation and the mechanisms by which it governs microglial polarization may pave the way for the development of novel therapeutic strategies for AD and other neuroinflammatory diseases.

#### 3.1.3. JAK/STAT signaling pathway

The JAK/STAT signaling pathway is integral in orchestrating immune and inflammatory responses, and has been implicated in the pathogenesis of neurodegenerative diseases, including AD (Ruganzu et al., 2021). Specifically, STAT1 and STAT3 have been identified as key players in mediating M1 microglial polarization by promoting the production of pro-inflammatory cytokines and chemokines (Lan et al., 2017). On the other hand, the activation of STAT6 by IL-4 is associated with the promotion of microglial polarization toward the M2 phenotype, a state considered to be neuroprotective due to its anti-inflammatory effects. This anti-inflammatory state is particularly significant as it could potentially mitigate the neuroinflammatory processes seen in AD (Lan et al., 2017). In terms of the mechanistic process, activation of the JAK/STAT pathway results in JAK-induced phosphorylation of STAT family proteins (Cianciulli et al., 2017). These phosphorylated STAT proteins then translocate to the nucleus and initiate the transcription of target genes, one of which includes the suppressor of cytokine signaling (SOCS) family (Xin et al., 2020). The SOCS family proteins serve as key negative feedback regulators, inhibiting the phosphorylation of JAK (Durham et al., 2019). Notably, numerous studies have shown that upregulated SOCS1 and SOCS3 could attenuate inflammatory responses and contribute to the mitigation of AD-associated neuroinflammation by promoting a shift in microglia from the M1 to M2 phenotype (Ruganzu et al., 2021). Therefore, these findings underscore the significance of the JAK/STAT pathway in the context of AD, by regulating microglial polarization and subsequent inflammatory responses. This intricate interplay between the JAK/STAT signaling pathway and microglial polarization offers potential therapeutic avenues for AD.

### 3.2. Anti-inflammatory (M2) polarization

#### 3.2.1. AMPK signaling pathway

Inflammatory responses are known to trigger an increase in intracellular Calcium (Ca^2+^) influx, which initiates a series of biochemical reactions crucial in the context of microglial activation and AD pathogenesis. Specifically, the increased Ca^2+^ influx binds to calmodulin (CaM), thereby activating the highly conserved Ca^2+^/CaM kinase cascade, including Calcium/calmodulin-dependent protein kinase kinase-β (CaMKKβ) (Marcelo et al., 2016; Dalal et al., 2020). Once activated, CaMKKβ phosphorylates AMP-activated protein kinase (AMPK), a central energy sensor in many tissues including the brain (Liu and Chern, 2015; Marcelo et al., 2016). It’s worth noting that AMPK plays a pivotal role in microglial polarization, particularly in shifting microglia from the pro-inflammatory M1 phenotype to the neuroprotective M2 phenotype (Wang J. et al., 2018). This process is particularly relevant in the context of AD, where a shift toward M2 phenotype could potentially mitigate neuroinflammation and slow disease progression.

In addition to CaMKKβ, liver kinase B1 (LKB1), another upstream kinase, is involved in phosphorylating and activating AMPK. This activation is prompted by an increase in the AMP/ATP ratio, a common occurrence during cellular stress situations such as neuroinflammation seen in AD (Deng et al., 2019). Subsequently, AMPK activates peroxisome proliferator-activated receptor γ coactivator 1α (PGC-1α) directly and also stimulates the production of NAD^+^, leading to the activation of Sirtuin 1 (Sirt1). Sirt1 then deacetylates and activates PGC-1α, a master regulator of mitochondrial biogenesis and function, whose dysregulation has been implicated in AD (Fernandez-Marcos and Auwerx, 2011). Originally, PGC-1α was identified through its interaction with peroxisome proliferator-activated receptor gamma (PPAR-γ), which consequently led to an increase in PPAR-γ transcriptional activity (Zhang et al., 2020). This process is significant, as PPAR-γ activation has been associated with beneficial effects in AD, including reduced neuroinflammation and Aβ burden.

#### 3.2.2. PI3K/AKT signaling pathway

The Serine and Threonine kinase AKT, also known as Protein Kinase B (PKB), plays a critical role in the regulation of microglial polarization (Manning and Toker, 2017). The activation of AKT is stimulated by G-protein-coupled receptors (GPCRs) or receptor tyrosine kinases (RTKs), culminating in the activation of phosphoinositide 3-kinase (PI3K). PI3K subsequently phosphorylates the T308 and S473 residues of AKT, leading to its full activation (Manning and Toker, 2017). Interestingly, low levels of PI3K/AKT signaling are associated with the pro-inflammatory M1 phenotype, while enhanced PI3K/AKT signaling is linked to the anti-inflammatory M2 phenotype (Linton et al., 2019). The PI3K/AKT pathway also maintains extensive interactions with other major signaling pathways, thereby influencing diverse cellular functions. For instance, it operates downstream of the NF-κB pathway, promotes neuroinflammatory responses in AD (Yu et al., 2017). Additionally, the activation of PI3K/AKT pathway is facilitated by phosphorylated Janus Kinase (JAK), another key modulator of immune responses (Gao et al., 2018). AKT can indirectly inhibit AMPK activation by promoting ATP production or directly phosphorylate AMPK, which inhibits LKB1-mediated phosphorylation of AMPK (Manning and Toker, 2017). This interaction is particularly noteworthy considering AMPK’s role in microglial polarization and AD pathogenesis.

Furthermore, the PI3K/AKT pathway and the extracellular signal-regulated kinase (ERK) pathway exhibit mutual inhibition, adding another layer of complexity to the regulation of cellular functions, including cell proliferation, differentiation, metabolism, and immune responses (Manning and Toker, 2017). In summary, the PI3K/AKT pathway’s intricate crosstalk with other signaling pathways and its role in microglial polarization underscores its potential as a therapeutic target in neuroinflammatory diseases such as AD. Further exploration of these molecular interactions may yield novel insights into AD pathogenesis and treatment strategies.

#### 3.2.3. PPAR-γ signaling pathway

Peroxisome proliferator-activated receptor-gamma (PPAR-γ), a ligand-inducible transcription factor belonging to the nuclear receptor superfamily, is significantly expressed in microglia and plays a pivotal role in AD pathogenesis (Carta and Pisanu, 2013). The regulatory function of PPAR-γ in microglia involves modulation of both pro- and anti-inflammatory cytokines and up-regulation of the M2 phenotype marker, Arginase-1 (Arg-1) (Ahmadian et al., 2013; Pisanu et al., 2014; Xu et al., 2015; Song et al., 2016; Song and Suk, 2017). Significantly, the activation of PPAR-γ signaling by Interleukin-4 (IL-4) drives microglial polarization toward the M2 phenotype (Jiang et al., 2020). Moreover, PPAR-γ serves as a crucial counter-regulatory mechanism, mitigating neuroinflammation by antagonizing the action of pro-inflammatory transcription factors such as AP-1, NF-κB, and STAT1 (Jacobi et al., 2012). For instance, PPAR-γ activation can disrupt the NF-κB signaling pathway by inhibiting the nuclear translocation of the p65 subunit or competing with NF-κB co-activators (Yang Z. et al., 2019). Interestingly, recent research has also shed light on the role of PPAR-γ in autophagy regulation in microglia. Studies indicate that PPAR-γ antagonism promotes the transition of microglia from M1 to M2 polarization by enhancing autophagy *via* the LKB1/AMPK signaling pathway (Ji et al., 2018). Given the involvement of impaired autophagy in the accumulation of Aβ and tau proteins, the role of PPAR-γ in autophagy regulation further underscores its potential as a therapeutic target in AD.

#### 3.2.4. Notch signaling pathway

The Notch signaling pathway is a highly conserved, cell-to-cell communication mechanism with fundamental roles in various cellular processes, including proliferation, differentiation, stem cell maintenance, and apoptosis (Nowell and Radtke, 2017). The pathway comprises four Notch receptors (NOTCH 1–4) and five ligands from the Delta-Serrate-Lag (DSL) family, including Jagged 1 (JAG1), JAG2, and Delta-like 1, 3, and 4 (DLL1, DLL3, DLL4). These Notch receptors are transmembrane proteins, synthesized in the endoplasmic reticulum and transported to the plasma membrane. In response to ligand binding from an adjacent cell, the Notch receptor undergoes a proteolytic cleavage, releasing the Notch intracellular domain (NICD) (Majumder et al., 2021). This NICD translocates into the nucleus where it acts as a transcriptional co-activator, inducing the expression of target genes and regulating cellular processes. In the context of microglia, the Notch signaling pathway has been recognized for its role in dictating microglial function and polarization (Wu et al., 2018). In AD, an imbalance in microglial polarization, favoring the pro-inflammatory M1 state, contributes to chronic neuroinflammation, exacerbating neuronal damage and disease progression. Notch signaling, through its influence on microglial polarization, might be involved in this process, possibly serving as a potential therapeutic target for rebalancing microglial states and mitigating neuroinflammation in AD. Furthermore, studies have suggested that Notch signaling might directly contribute to AD pathology, with aberrant Notch activation observed in AD models and patient samples. This aberrant activation might influence the production and clearance of Aβ plaques.

### 3.3. The crucial role of P2 × 7 receptor in microglial M1/M2 phenotypic balance and AD progression

Microglia showcase a diverse range of activation states rather than being exclusively confined to the traditionally defined M1 and M2 phenotypes. This nuanced view is substantiated by recent transcriptomic analyses that illuminate the existence of several intermediate phenotypes bridging the classical M1 and M2 states (Ransohoff, 2016). Moreover, microglia have the ability to concurrently demonstrate both pro-inflammatory and phagocytic functions, indicating that these two functions are not mutually exclusive but may interact dynamically (Masuda et al., 2020). Within this context, the P2 × 7 receptor (P2 × 7R), a purinergic receptor abundantly expressed in microglia, plays a pivotal role. Intriguingly, P2 × 7R has been observed to bolster phagocytosis even in the absence of an external ATP agonist (Gu et al., 2010, 2011, 2012; Gu and Wiley, 2018). This unique function is seemingly regulated by the closed state of the receptor and involves residues 306–320 of P2 × 7R binding to extracellular debris, bacteria, and apoptotic cells (Gu et al., 2011). Moreover, it includes interactions of intracellular regions of P2 × 7R with non-muscle myosin heavy chain IIA (NMMHC IIA) and the cytoskeleton, which in turn facilitate the internalization of material into the cell (Gu et al., 2010, 2009). This phagocytic capacity can be hindered by cytochalasin D or a monoclonal antibody targeting the extracellular domain of P2 × 7R, emphasizing the receptor’s indispensable role in this process. Furthermore, the synthesis and surface expression of P2 × 7R were identified as crucial for the phagocytic function of human monocytic cells, and the interruption of these processes by siRNA led to a marked impairment of phagocytic function (Gu et al., 2011).

When extracellular ATP levels surge transiently, the P2 × 7 receptor transitions from acting as a scavenger receptor. This triggers an upswing in autophagy, indicated by a transient elevation in levels of the autophagosomal lipid marker LC3-II, resulting in the degradation of materials and pushing microglia toward a mixed M1/M2 activation state (Gu and Wiley, 2018; Campagno and Mitchell, 2021). The dynamic equilibrium between these M1 and M2 states is pivotal in microglia’s transformation into disease-associated microglia (DAM), a distinct state seen in neurodegenerative diseases like AD (Orre et al., 2014; Keren-Shaul et al., 2017). As DAMs recognize neurodegeneration-associated molecular patterns (NAMPs) such as Aβ plaques, they are believed to exhibit a hybrid M1/M2 phenotype (Deczkowska et al., 2018). Emerging evidence suggests that P2 × 7R’s modulation of the M1/M2 balance in microglia may impact the onset and progression of the DAM state, thereby potentially influencing the course of neurodegenerative diseases (Illes et al., 2020). Considering the complex roles P2 × 7R plays in molding microglial phenotypes and DAM states, P2 × 7R antagonists have displayed promise in ameliorating various neurodegenerative conditions (Beamer et al., 2017; Fabbrizio et al., 2017; Wang et al., 2017; Domercq and Matute, 2019; Illes et al., 2019a). Notably, selective P2 × 7R antagonists can block the receptor’s channel activity without disrupting its phagocytic function, offering a potential mechanism to restore a balance between inflammation and phagocytosis (Fletcher et al., 2019).

Under pathological conditions, persistent ATP stimulation activates P2 × 7R (Burnstock et al., 2011; Li et al., 2015; Illes et al., 2019b). Within an inflammatory milieu (M1 phenotype), this activation prompts a cascade of cellular responses, beginning with the interaction of Toll-Like Receptor 4 (TLR4) with lipopolysaccharide (LPS) and culminating in ATP-dependent stimulation of P2 × 7R. This interaction subsequently instigates NLRP3 inflammasome-mediated caspase-1 activation and secretion of the pro-inflammatory cytokine IL-1β (Perregaux and Gabel, 1998; Muñoz-Planillo et al., 2013). This process is primarily stimulated by a decrease in intracellular potassium levels, illustrating the intricate ion regulatory role of P2 × 7R (Di Virgilio et al., 2017, 2018; Madry et al., 2018). P2 × 7R activation can also lead to the release of other pro-inflammatory cytokines, such as IL-6 and TNF-α, further accentuating the inflammatory state (Shieh et al., 2014).

**FIGURE 2:**
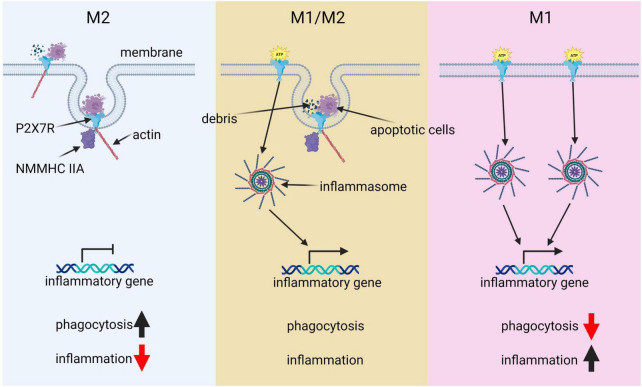
The pivotal role of P2 × 7R in microglial polarization. This diagram illustrates the key role of P2 × 7R in shaping microglial polarization states and its influence on immune responses. Under normal conditions, P2 × 7R interacts with extracellular debris such as apoptotic cells, as well as intracellular cytoskeleton components like actin and NMMHCIIA. This interaction induces phagocytic activity, resulting in minimal inflammation and predominance of the M2 state. However, transient ATP stimulation alters this balance: P2 × 7R not only drives phagocytosis but also gets activated by ATP, triggering downstream immunological inflammasome pathways and promoting inflammatory responses, thereby leading to a balance between M1 and M2 states. In a pathological condition, chronic ATP binding and activation of P2 × 7R shifts the cell primarily toward a pro-inflammatory and phagocytosis-suppressed phenotype, characteristic of the M1 state.

In summary, microglia present a complex activation spectrum that extends beyond the traditional M1 and M2 phenotypes, exhibiting multiple intermediate states (Figure 2). P2 × 7R significantly influences this phenotypic diversity by coordinating pro-inflammatory and phagocytic responses. Further, P2 × 7R is implicated in the progression of DAM states, notably in neurodegenerative conditions. Understanding the intricate role of P2 × 7R in managing microglial phenotypes and its therapeutic potential necessitates further research to develop effective strategies for combating AD.

## 4. Phagocytosis of microglia in AD

Microglia play a pivotal role in the pathogenesis of AD by phagocytosing various targets such as apoptotic neurons, bacteria, lipoproteins, and Aβ (Hansen et al., 2018). The effect of microglia on AD progression depends on the specific substrate being phagocytosed. Microglial phagocytosis of Aβ is essential for clearing Aβ deposits and is considered a neuroprotective mechanism (Ries and Sastre, 2016). Microglia engulf Aβ through various receptors, such as Tyro3/Axl/Mer receptor tyrosine kinases (TAM) (Huang et al., 2021), scavenger receptor A (SRA), CD36 (Frenkel et al., 2013; Lucin et al., 2013), transporting it to lysosomes, where it is compressed into dense core plaques and toxic substances. Overactivation or phagocytic dysfunction of microglia can lead to Aβ accumulation and promote AD progression. Excessive phagocytosis of normal neuronal synapses by microglia is a critical factor contributing to cognitive decline in AD. Aβ binding to complement component C1q can activate the classical complement pathway, resulting in excessive microglial phagocytosis of synapses. This complement-dependent pathway and excessive microglial activation, leading to synaptic loss, are associated with cognitive dysfunction in AD (Schafer et al., 2012). Inhibiting C1q, C3, and C3R expression can effectively reduce the number of activated microglia and early synaptic loss (Hong et al., 2016). Furthermore, microglial phagocytosis of the extracellular matrix (ECM) surrounding synapses promotes synaptic structural remodeling and is involved in memory consolidation. IL-33 mediates experience-dependent neuron-microglia communication, promoting hippocampal dendritic spine formation, synaptic remodeling, and ECM phagocytosis, all essential for memory consolidation. Loss of IL-33 results in impaired microglial phagocytosis of ECM, accompanied by the accumulation of ECM proteins related to synapses (Végh et al., 2014b; Nguyen et al., 2020). Clearing ECM and administering exogenous recombinant IL-33 have been shown to improve AD model mice (Végh et al., 2014a; Fu A. K. Y. et al., 2016). In this review, we will provide a comprehensive analysis of the genes involved in microglial phagocytic function, their implications for AD pathogenesis, and potential therapeutic targets.

### 4.1. Phagocytosis of apoptotic neurons by microglia

**FIGURE 3:**
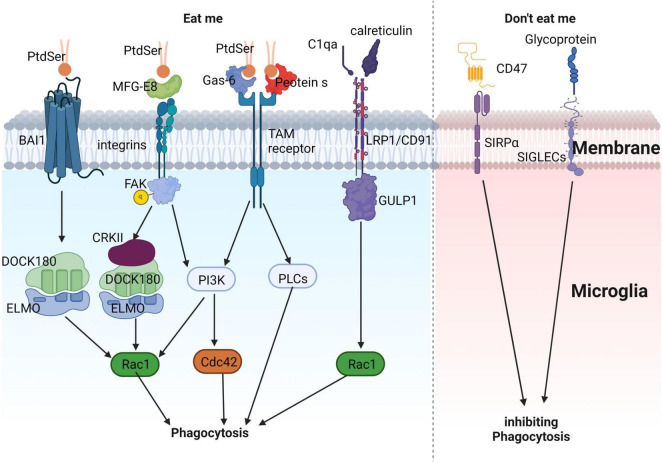
Illustrates the process of microglial phagocytosis of apoptotic neurons. The detection of dying neurons by microglia is mediated by the release of “eat-me” signals from apoptotic neurons. PtdSer interacts either directly with the microglial *BAI1* receptor or indirectly through MFG-E8, Gas-6, and protein S. These proteins can engage microglial receptors such as integrins and TAM receptors, ultimately promoting the phagocytosis of apoptotic neurons. Apart from PtdSer, other eat-me signals include calreticulin and complement component C1q. These molecules can associate with LRP1/CD91 to facilitate phagocytosis. In contrast, “don’t-eat-me” signals, which inhibit phagocytosis, consist of sialylated glycoproteins and lipids, as well as the CD47 protein. These components can interact with microglial receptors like SIGLECs and SIRPα, respectively, to suppress phagocytosis, ensuring a fine-tuned regulation of this crucial process.

In AD, microglia rapidly engulf dying or apoptotic neurons, suppressing further inflammation (Lazdon et al., 2020). The phagocytic process of microglia can be divided into three stages: “Find me,” “Eat me,” and “Digest me.” “Find me” signals are cell factors released by apoptotic cells that attract phagocytic cells. These signals include CX3CR1, LPC, S1P, and nucleotides, which bind to corresponding receptors on phagocytic cells (CX3CR1, G2A, S1P-R1/5, and P2Y2, respectively), promoting their migration toward apoptotic cells. “Eat me” signals are recognized by phagocytes following “Find me” signals. During phagocytosis, apoptotic cells expose “Eat me” signals, enabling phagocytes to distinguish them from healthy cells. Phosphatidylserine (PtdSer) is a crucial “Eat me” signal, which can directly or indirectly bind to phagocyte surface receptors and mediate downstream signaling pathways (Arandjelovic and Ravichandran, 2015; Figure 3). These include: (1) direct binding of PtdSer to PtdSer receptors such as BAI1, which recognizes PtdSer and binds to intracellular ELMO1 and Dock, activating downstream Rac1, causing actin and other cytoskeleton rearrangements, and promoting phagocytosis of apoptotic cells (Arandjelovic and Ravichandran, 2015); (2) PtdSer activates integrin receptors through bridging molecules such as MFG-E8, and activates downstream Rac1 *via* the CrkII-Dock180-ELMO signaling pathway (Arandjelovic and Ravichandran, 2015); (3) PtdSer activates TAM receptors through bridging molecules such as Gas6 or Protein S, and downstream signaling pathways may induce cytoskeleton remodeling by activating PI3K or PLC (DuBois et al., 2020; Lew et al., 2020; Rosin et al., 2021); and (4) in addition to PtdSer, calcium-binding protein (calreticulin) or complement C1qa can also activate LRP1/CD91 to bind to scaffold protein GULP1 and activate downstream Rac1 (Cockram et al., 2019; Hammond et al., 2020). “Digest me” signals mediate the processing of phagocytosed apoptotic cells, which requires the maturation of phagosomes (Han et al., 2021).

”Don’t eat me” signals inhibit phagocytic cells from exerting their phagocytic function. CD47, a transmembrane protein expressed in most mammalian cells, including neurons, inhibits phagocytic activity *via* the signal-regulatory protein alpha (SIRPα) on phagocyte surfaces. CD47 also inhibits microglial-mediated synapse clearance during development (Butler et al., 2021). Sialic acid proteins inhibit neuronal phagocytosis by activating sialic acid-binding immunoglobulin-like lectins (SIGLECs) on microglial surfaces (Brown and Neher, 2014).

Additionally, a novel phagocytic pathway called LC3-associated phagocytosis (LAP) has recently been reported. LAP plays a role in engulfing both apoptotic and live cells. Injecting apoptotic cells into LAP-deficient animals promotes lupus-like disease, increases pro-inflammatory cytokine production, and decreases anti-inflammatory cytokine production (Martinez et al., 2016). LAP inhibits the inflammatory response during engulfment of apoptotic cells *in vitro* and *in vivo*. During LAP, the engulfment body recruits components of the autophagic machinery, catalyzes the lipidation of LC3 on the engulfment body membrane into phosphatidylethanolamine, and induces engulfment body maturation and cargo degradation. LAP is induced by binding of TLR1/2, TLR2/6, or TLR4, Fc receptors, and apoptotic cell receptor TIM4 on the engulfment body (Heckmann et al., 2017). Unlike canonical autophagy, LAP does not require components of the preinitiation complex (ULK1, FIP200, ATG13) to initiate autophagy in response to nutrient stress, but it requires a unique Beclin-1 (BECN1) and VPS34 initiation complex that lacks ATG14 but includes Rubicon (Heckmann et al., 2017). Phagocytosis and clearance of substrates are important pathways for microglia to maintain tissue homeostasis.

#### 4.1.1. C1q: facilitator of microglial phagocytosis and synaptic pruning in AD

C1q is a critical protein that regulates the production of inflammatory cytokines and aids in the removal of apoptotic neurons and neuronal blebs by microglia. Direct binding to Aβ and being the initial constituent of the classical complement pathway are among the functions of C1q (Jiang et al., 1994). However, complement activation can lead to neuronal lysis, resulting in inflammation, neuronal damage, and impaired neuronal integrity (Tenner, 2001). C1q’s involvement in the initiation of the classical complement pathway results in several downstream effects, including opsonin C3b deposition on target cell surfaces, pathogen lysis, and phagocytic cell recruitment (Fraser and Tenner, 2008). During the early stages of cell death, C1q’s role in the central nervous system is protective, increasing microglial clearance of apoptotic cells and suppressing proinflammatory cytokines (Fraser et al., 2010). However, in AD pathology, C1q’s role is more complex, with associations with both fibrillar Aβ plaques and tangles (Afagh et al., 1996; Shen et al., 2001). C1q positive Aβ plaques are associated with reactive astrocytes or microglia, which are frequently associated with the neurodegenerative process (Dickson, 1997; Griffin et al., 1998). Increased recruitment of activated glial cells may cause enhanced inflammation and contribute to the relationship between C1q and AD pathology (Fonseca et al., 2004a). Furthermore, C1q enhances phagocytosis and binding to apoptotic cells, which is a complement and microglia-dependent pathway that removes excess synapses, overactivation of this pathway leads to synaptic loss in AD (Korb and Ahearn, 1997; Webster et al., 2000; Hong et al., 2016). C1q inhibition decreases the number of phagocytic microglia and the amount of initial synapse loss by inhibiting C1q, C3, or the microglial complement receptor CR3 (Fonseca et al., 2004b; Hong et al., 2016; Shi et al., 2017). C1q is believed to exert toxic effects on hippocampal long-term potentiation (LTP) and synapses affected by soluble Aβ (Hong et al., 2016). In summary, C1q’s role in AD pathology is complex, with both protective and harmful effects.

#### 4.1.2. TLR-4: essential modulators of microglial phagocytosis and axon clearance in the CNS

Studies have shown that the phagocytosis of myelin by microglia and the clearance of apoptotic cells contribute significantly to the inhibition of inflammation and restoration of the CNS (Fadok et al., 1998; Franklin and Kotter, 2008). TLRs also play a critical role in the clearance of oligomeric, monomeric, and fibrillar Aβ by microglia (Reed-Geaghan et al., 2009). Additionally, TLRs such as TREM2, PtdSer receptor T cell immunoglobulin mucin-4, and soluble linking proteins MFG-E8 and Gas6 are crucial for microglial phagocytosis of apoptotic cells (Miyanishi et al., 2007; Grommes et al., 2008; Linnartz and Neumann, 2013). Genetic and pharmacological interruption of TLR4 blocks the induction of the type-1 interferon response, restricting the phagocytosis of ex-vitro axon remains, while TLR4-dependent microglial clearance of unmyelinated axon remains facilitates the outgrowth of axons (Takahashi et al., 2005; Rajbhandari et al., 2014). TLR4-mediated signaling may also be affected by purinergic receptors such as the P2 × 7 receptor (P2 × 7R), which may lead to the clearance of microglia-degenerated axons (Takahashi et al., 2005; Rajbhandari et al., 2014). Elimination of TLR4 in adult mice results in impaired microglial phagocytosis of CNS axons, particularly neurons undergoing Wallerian degeneration in a model of dorsal root axotomy (Rajbhandari et al., 2014). These findings suggest that TLRs and purinergic receptors play an essential role in microglial phagocytosis and axon clearance, which may contribute to the restoration of the CNS.

#### 4.1.3. ANXA1: regulator of microglial phagocytosis and blood-brain barrier integrity

ANXA1 is a mediator of glucocorticoid anti-inflammatory effects in the peripheral system, contributing to the efficient elimination of apoptotic neuron-form cells (Perretti and D’Acquisto, 2009; McArthur et al., 2010). It also plays a crucial role in maintaining the tightness of the blood-brain barrier (BBB) by being present in the BBB endothelium and microglial cells (Solito et al., 2008; Cristante et al., 2013). Microglia can synthesize and release ANXA1, which is associated with regulating leukocyte extra-vasation, phagocytosis, and glucocorticoid actions (Young et al., 1999; Perretti et al., 2002; Yona et al., 2004; Buckingham et al., 2006). ANXA1 can be upregulated in human microglia surrounding Aβ plaques and has been identified as a receptor for the formyl peptide receptor-like 1 (FPRL1), which also interacts with Aβ (Young et al., 1999; Buckingham et al., 2006). The attachment of ANXA1 to FPRL1 regulates microglial phagocytosis and proinflammatory cytokine release, enhancing the phagocytic action of microglia (Young et al., 1999; Le et al., 2001; Pan et al., 2011). Therefore, ANXA1 is a critical factor in maintaining the integrity of the BBB and regulating microglial function, which may have implications for neuroinflammatory conditions.

### 4.2. Trogocytosis of microglia

Trogocytosis is a process by which microglia nibble off portions of other live cells, primarily synapses, unlike phagocytosis, which targets dead cells (Weinhard et al., 2018). Microglia use trogocytosis to reshape neuronal synapses, mediating synaptic remodeling (Lim and Ruthazer, 2021). While the molecular mechanisms of trogocytosis have been well-studied in peripheral immune cells, recent research has shed light on the role of trogocytosis in microglia. The intercellular receptor-ligand interaction is crucial for trogocytosis, and actin and PI3K are involved (Bettadapur et al., 2020). In the immune system, trogocytosis is associated with the downregulation of the “Don’t eat me” signal CD47-SIRPα and the upregulation of the CD11b/CD18 integrin pathway (Matlung et al., 2018). Receptors play a critical role in determining the activation of immune cells in phagocytosis or trogocytosis. Chimeric antigen receptor-phagocytic receptors (CAR-Ps) can guide macrophages to ingest target cells through trogocytosis rather than phagocytosis, and this process depends on the ITAM-containing intracellular signaling domain (Morrissey et al., 2018). The specificity of receptor interaction plays an important role in mediating phagocytosis or trogocytosis. Conflicting results exist regarding the role of the complement pathway in microglial trogocytosis. The absence of CR3 did not significantly affect trogocytosis in microglia *in vitro* brain slice cultures of CR3 knockout mice, suggesting that complement pathway participation is not required for microglial trogocytosis (Weinhard et al., 2018). However, *in vivo* studies in African clawed frogs indicate that trogocytosis in microglia required the complement pathway, and C3 mediate microglial trogocytosis (Hodge et al., 2019; Lim and Ruthazer, 2021). Further research on the molecular mechanisms of trogocytosis in different cell types and pathological states is necessary to better understand this unique process and develop potential therapeutic strategies.

### 4.3. Comparation between phagocytosis and trogocytosis

**FIGURE 4:**
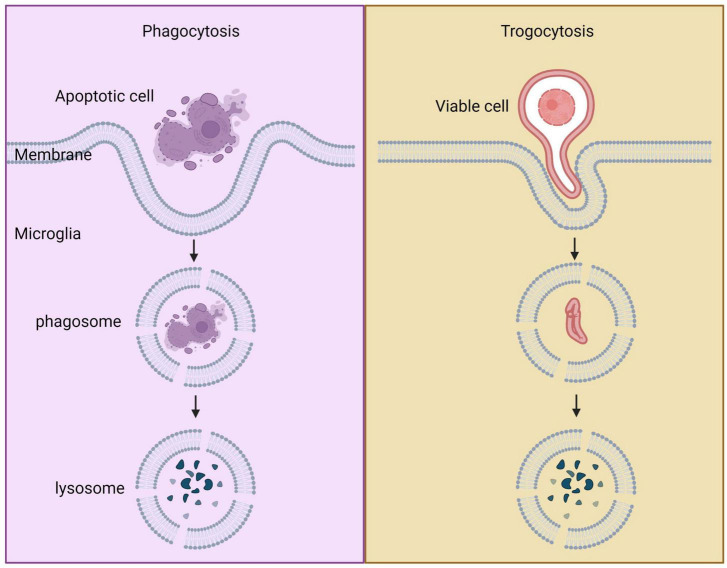
Comparison between phagocytosis and trogocytosis. Trogocytosis is a process where live cell targets are nibbled, whereas phagocytosis is a process where dead cell targets are engulfed.

In organisms, phagocytosis and trogocytosis are two primary ways that macrophages clear cells or particles. Phagocytosis is used for larger particles, such as dead cells, and involves engulfing the entire target. Trogocytosis, on the other hand, is used for smaller particles, generally less than 1 μm in size, and involves nibbling away at the target (Figure 4). If the target is too large to be internalized through phagocytosis, macrophages will surround the target, which is known as “phagocytic failure” (Chen et al., 2019). It’s important to note that macrophages involved in phagocytic failure do not digest the target through nibbling, so trogocytosis is not the result of phagocytic failure. The molecular mechanisms underlying phagocytosis and trogocytosis are also different. During phagocytosis, highly conserved pathogen-associated molecular patterns (PAMP) bind to FcγR and enter the cell, activating downstream signaling pathways that induce actin polymerization and formation of phagosomes. When FcγR binds to artificially designed antibodies, it can induce macrophages that originally have phagocytic activity to take up target cells through trogocytosis and induce non-professional phagocytic cells to exhibit trogocytic function (Morrissey et al., 2018). In the CNS, microglia are responsible for phagocytosing neuronal synapses (Schafer et al., 2012). The complement pathway is activated during this process, with C1q binding to and opsonizing apoptotic cells, pathogens, or debris. This triggers a cascade of proteases that result in the deposition of downstream complement protein C3. C3 deposited on dendrites and synapses can directly activate C3 receptors on microglia, leading to dendrite and synapse phagocytosis. The key step in complement pathway activation is the cleavage of C3 into C3a and C3b, where C3a recruits and activates microglia, and C3b opsonizes neurons and synapses (Butler et al., 2021). Studies have shown that excessive phagocytosis of weak synapses by complement-mediated microglia may lead to cognitive impairment during memory extinction (Butler et al., 2021). Conversely, knocking out C1q, C3, or CR3 in mice reduces synaptic pruning during development, leading to the production of excess synapses (Hong et al., 2016). Research on the molecular mechanisms underlying microglial trogocytosis in the CNS is still incomplete. It is unclear whether the complement pathway is involved in microglial trogocytosis. Further investigation into the molecular mechanisms of trogocytosis is of great significance for understanding the pathogenesis of neurodegenerative diseases and may provide new molecular targets for their treatment.

### 4.4. Phagocytosis of Aβ

Current research has focused on understanding how Aβ is removed from the body. Aβ can enter peripheral circulation through various routes, such as transfer across the blood-brain barrier, the glymphatic system, or perivascular drainage (Shibata et al., 2000; Weller et al., 2008; Iliff et al., 2012; Iliff and Nedergaard, 2013). Microglia cells are known to play a role in phagocytosis of Aβ, including soluble oligomeric Aβ. Other cells, such as, astrocytes, olfactory ensheathing cells (OECs), and neurons, also contribute to degrading and clearing Aβ (Scheuner et al., 1996; Malm et al., 2010). Certain molecules, such as MFG-E8 and the TAM receptor family, aid in the phagocytosis process by binding to Aβ. However, the way in which Aβ aggregates can vary depending on the binding proteins and their interaction with Aβ.

#### 4.4.1. MFG-E8: promotor of microglial phagocytosis of Aβ

MFG-E8 is a vital molecule that plays a crucial role in the process of phagocytosis, which involves the engulfment and elimination of apoptotic cells. This molecule binds to integrin molecules such as integrin β3 on the surface of phagocytes. Integrin receptors consist of paired α and β sub-units (Hynes, 2002; Barczyk et al., 2010) and are essential for various cellular functions such as adhesion, migration, growth, differentiation, and reprogramming in macrophages (Chen et al., 2009; Barczyk et al., 2010). Produced by macrophages, MFG-E8 helps to alleviate inflammation and promote efferocytosis, which is the process of clearing apoptotic cells (Kojima et al., 2017). Interestingly, there appears to be a direct link between MFG-E8 and transglutaminase 2 (TG2), which is also involved in cholesterol transfer and enhances phagocytosis (Hanayama et al., 2002; Boisvert et al., 2006). TG2 acts as a co-receptor of β3 integrin and attaches to extracellular MFG-E8 (Tóth et al., 2009). Recent studies have shown that microglia and astrocytes release MFG-E8 in response to stimuli from neurons triggered by Aβ (Neher et al., 2011; Kawabe et al., 2018). TG2 also contributes to phagocytosis, with research indicating that TG2 peritoneal macrophages play an essential role in recognizing apoptotic cells through MFG-E8 and integrin β3 (Akimov et al., 2000; Tóth et al., 2009). Furthermore, the TG2 protein mediates the attachment between MFG-E8 and the vitronectin receptor (VR) independently of TG enzyme activity (Tóth et al., 2009; Kawabe et al., 2018). Studies have shown that MFG-E8 reduces cell death in response to Aβ-induced neurons and enhances microglial phagocytosis of Aβ. MFG-E8 related therapy also activates the nuclear factor erythroid 2-associated factor 2 (Nrf2)-HO-1 pathway, which protects Aβ-activated neurons from cell death (Li et al., 2012). In summary, MFG-E8 is a critical molecule involved in phagocytosis and is produced by macrophages in response to inflammation. Its interaction with integrin molecules and TG2 contributes to the recognition of apoptotic cells and promotes their clearance. In the brain, MFG-E8 plays a crucial role in reducing neuronal cell death and enhancing microglial phagocytosis of Aβ, suggesting that it could be a potential therapeutic target for AD.

#### 4.4.2. CD36: crucial for fibrillar Aβ phagocytosis but non-essential for soluble Aβ clearance

CD36, a member of the scavenger receptor class B family, is expressed on the surface of various cells such as neurons, macrophages, astrocytes, and monocytes (Yu and Ye, 2015). It plays an important role in the phagocytosis of fibrillar Aβ_42_ by binding to it (Wilkinson and El Khoury, 2012; Yu and Ye, 2015). Studies have shown that CD36 deficiency in mice prevents the accumulation of microglia in response to the injection of fibrillar Aβ (El Khoury et al., 2003), indicating that intracerebral microinjection of fibrillar Aβ can be used to study the molecular and cellular mechanisms of Aβ-mediated brain responses and screen for new therapies in CD36 knockout mice (El Khoury et al., 2003). *In vitro* experiments have also shown that antagonists of CD36 can efficiently block the phagocytosis of fibrillar Aβ_42_ by microglia (Koenigsknecht and Landreth, 2004). The expression of CD36 is significantly higher in monocyte-extracted macrophages activated by glatiramer acetate, which plays an important role in the clearance of Aβ (Koronyo et al., 2015). While CD36 can bind to soluble Aβ42, its role in the clearance of soluble Aβ is not essential (Frenkel et al., 2013; Sheedy et al., 2013). Knockdown or suppression of CD36 does not affect the continuous capability of microglia to phagocytose soluble Aβ_42_. The prolonged expression of additional scavenger receptors compensates for the loss of CD36 function (Wilkinson et al., 2011). However, the deficiency of class A1 scavenger receptors impairs the clearing of soluble Aβ *via* mononuclear phagocytes and accelerates the development of AD (Frenkel et al., 2013). Interestingly, the selective elimination of CD36 does not affect the uptake of soluble Aβ, suggesting that CD36 is not necessary for clearing soluble Aβ. However, CD36 is involved in activating mononuclear phagocytes through soluble Aβ, which may lead to the production of cytokines and reactive oxygen species (El Khoury et al., 2003; Stewart et al., 2010; Frenkel et al., 2013). This is similar to the role CD36 plays in the interaction between oxidized low-density lipoproteins and macrophages (Maxeiner et al., 1998).

#### 4.4.3. LRP1: facilitators of Aβ clearance in AD pathology

Recent studies suggest that LDL-receptor-associated protein 1 (LRP1) plays a crucial role in discriminating between apoptotic and normal cells. Up-regulation of calreticulin (Calr) on the apoptotic cell surface enhances their interaction with LRP1 and promotes the activation of phagocytic cells, leading to successful engulfment of apoptotic cells (Gardai et al., 2005). LRP1 forms a complex with Calr, functioning as a collectin receptor on macrophages. The role of LRP1 has also been demonstrated in the clearance of Aβ and transmission of signals involved in the pathology of AD (Kanekiyo and Bu, 2014). However, expression of *APOE4* can lead to less efficient microglial clearance of Aβ, thereby affecting Aβ metabolism and microglial response (Zhao et al., 2014; Krasemann et al., 2017). *APOE* isoforms compete for identical clearance paths into the brain and can affect Aβ metabolism, as evidenced by studies in *APOE*-deficient mice exhibiting reduced fibrillar plaque deposition and altered regional distribution of plaque pathology within the hippocampus (Verghese et al., 2013; Ulrich et al., 2018). In a mouse model, *APOE* isoforms modulate the metabolism of soluble amyloid beta protein (sAβ) within astrocytes and the interstitial fluid by competing for the LRP1-dependent cellular absorption pathway in astrocytes (Verghese et al., 2013). Additionally, *APOJ* modifies the capability of forming fibrils and alters Aβ-induced neurotoxicity. Both *APOE* and *APOJ* are generated in astrocytes, where they facilitate the transport and clearance of Aβ through the blood-brain barrier *via* the megalin/LRP-2 receptor. Exposure of astrocytes to Aβ fused with *APOJ*, α1-antichymotrypsin (ACT), *APOE*, and integration of serum amyloid protein (SAP) and supplement C1q leads to a significant decrease in astrocyte expression, while no microglial Aβ absorption is observed. These findings highlight the crucial role of LRP1 and *APOE* isoforms in the clearance of Aβ and the development of AD pathology (Mulder et al., 2014).

#### 4.4.4. ABCA7: enhancer of Aβ clearance in AD pathology

ABCA7 is a cytosolic protein that plays a crucial role in phagocytosis, and its upregulation has been associated with increased phagocytosis in certain disorders (Jehle et al., 2006; Yancey et al., 2010). Recent genome-wide studies have revealed a significant relationship between ABCA7 and AD (Hollingworth et al., 2011). This transporter is predominantly expressed in microglia, where it protects the brain by regulating the secretion and deposition of Aβ (Kaminski et al., 2000; Koldamova et al., 2003; Wahrle et al., 2005). Studies have demonstrated that ABCA7^–/–^ mice exhibit reduced phagocytic clearance of Aβ oligomers in the hippocampus. Moreover, upregulation of ABCA7 in the hippocampus of the AD brain and amyloidogenic mouse brain appears to contribute to the pathogenesis of Aβ (Fu Y. et al., 2016).

#### 4.4.5. TNF-α: influencing microglial phagocytosis and Aβ production in AD

The link between neuroinflammation and AD pathogenesis is well-established through clinical and experimental evidence (Heneka et al., 2015; Heppner et al., 2015). Inflammatory molecules such as IL-6, IL-1β, and TNF-α have been implicated in AD pathology (Wyss-Coray and Rogers, 2012). TNF-α is known to play a critical role in AD pathogenesis as it is expressed in neurodegenerative diseases. Studies in 5 × FAD/*TNF*-α^–/–^ mice show that the absence of TNF-α reduces Aβ in the brain. TNF-α influences Aβ production by decreasing the levels of β- and α-secretases in 5 × FAD/*TNF*-α^–/–^ brains, and it also affects PS1-mediated activities by decreasing the levels of PS1-carboxyterminal particles. TNF-α defects lead to a decline in microglial and astrocyte activation, resulting in decreased phagocytic activities of macrophages, including responsiveness toward Aβ. Genetic deletion of *TNF*-α in 5 × FAD mice reduces amyloid plaque formation by reducing the production of Aβ through activation of PS1 and β-secretase, rather than enhancing Aβ clearance through phagocytosis. Paouri et al. showed that amyloid pathology was modulated by TNF-α using cells and glial from AD mice’s brain (Paouri et al., 2017a,b).

#### 4.4.6. γ-secretase: dual role in Aβ production and impaired phagocytosis in AD

γ-secretase is an enzyme that plays a critical role in the development of AD. Alterations in gamma-secretase activity can affect the expression of scavenger receptors, including SR-A, CD36, and CD40, which are involved in the clearance of Aβ by microglia (Husemann et al., 2002; El Khoury et al., 2003; Yang et al., 2011). While microglia and macrophages share similar phagocytic capabilities, blood-derived microglia have been shown to exhibit greater phagocytic activity toward Aβ deposits (Simard et al., 2006). Presenilin 1 and 2 (PS1 and PS2) are important determinants of gamma-secretase catalytic localization and play a crucial role in APP processing to generate neurotoxic Aβ isoforms (Wolfe, 2008). Depletion of the PS1 and PS2 catalytic site leads to impaired soluble Aβ phagocytosis and clearance of insoluble Aβ plaques. PS2 deficiency has been shown to impair Aβ phagocytosis in peritoneal macrophages *in vivo*. Furthermore, γ-secretase has a dual role in AD, as cleavage of APP by γ-secretase is associated with the pathogenesis of AD and Aβ accumulation, while microglial activation in AD is mediated by γ-secretase. Mutations in PS alter γ-secretase activity, leading to microglial dysfunction and increased amyloid aggregation in AD (Farfara et al., 2011). Therefore, dysfunction in microglial clearance of Aβ may be linked to alterations in γ-secretase activity, particularly in the context of PS mutations that accelerate AD pathology.

#### 4.4.7. RAGE: facilitator of Aβ-induced neurodegeneration and modulator of phagocytosis in AD

The receptor for advanced glycation end products (RAGE) is involved in the process of phagocytosis by acting as a receptor for PtdSer and may play a role in inflammation resolution (Friggeri et al., 2011; He et al., 2011). Additionally, RAGE can exist in a soluble form (sRAGE) that acts as a RAGE ligand (Santilli et al., 2009). RAGE can bind to the globular heads of C1q and enhance the phagocytosis of apoptotic cells mediated by C1q by forming a receptor complex with complement receptor 3 (CR3; CD11b/CD18) (Ma et al., 2012). When Aβ fibrils bind to RAGE, signal transduction is initiated through adaptor proteins on the cytoplasmic domain of RAGE (Yan et al., 1996). Oligomeric Aβ strongly activates RAGE in neurons and microglia, which leads to neurodegeneration (Yan et al., 2009; Origlia et al., 2010; Srikanth et al., 2011). Moreover, it has been suggested that scavenger receptor and macrophage receptor with collagenous structure (SR-MARCO) and RAGE form a network with FPRL1/FPR2 to initiate microglial signaling in response to Aβ (Slowik et al., 2012).

### 4.5. Phagocytosis of tau

Abnormal accumulation of hyper-phosphorylated tau protein contributes to neuronal death in AD (Medina and Avila, 2014). The CX3CL1/CX3CR1 signaling pathway is crucial in deactivating microglia and inhibiting the release of proinflammatory cytokines (Cardona et al., 2006; Biber et al., 2007; Ginhoux et al., 2010). Defects in this pathway result in the enhanced generation of proinflammatory molecules, which leads to the deterioration of Tau *via* microglia (Bolós et al., 2017). *In vitro* and *in vivo* studies have demonstrated that CX3CR1/CX3CL1 axis plays a significant role in phagocytosing Tau in microglia and is a contributing factor in AD development. Targeting CX3CR1 could be a potential strategy for clearing extracellular Tau (Bolós et al., 2017).

## 5. Inflammation

### 5.1. Neuroinflammation mediated by microglia in CNS

Neuroinflammation is the response of the CNS to endogenous and/or exogenous factors that disrupt normal cellular homeostasis (Maccioni et al., 2018). Microglia are essential for detecting various endogenous and exogenous signals in the CNS. Inflammatory signals are a category of stimuli that are classified as either danger-associated molecular patterns (DAMPs), pathogen-associated molecular patterns (PAMPs), or molecular processes that alter homeostasis (Schafer et al., 2012). Microglia are also involved in the entire process from innate immunity to subsequent inflammation in the brain (Maccioni et al., 2018; Pang et al., 2022; Van Zeller et al., 2022). Pattern recognition receptors (PRRs), such as TLRs, nucleotide-binding oligomerization domain (NOD)-like receptors (NLRs), and AIM2-like receptors, are involved in the detection of DAMPs and PAMPs by microglia in the CNS (Freeman et al., 2017; Ma et al., 2021). Following detection of DAMPs or PAMPs, microglia become activated and release a cascade of cytokines and chemokines in response to the injury or pathological event, which serves to elicit an immune response and modulate the microenvironment (Wang Y.-J. et al., 2021).

**FIGURE 5:**
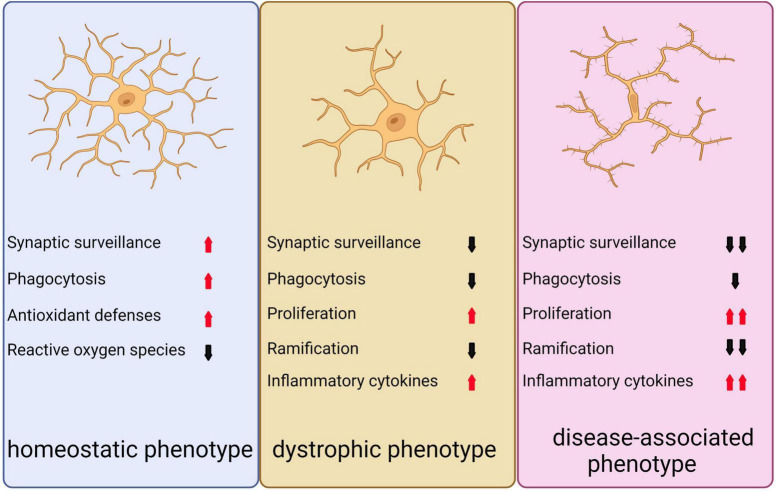
Depicts the three distinct microglial phenotypes. The first, known as the homeostatic phenotype, is generally observed in the adult brain under non-pathological, non-infected, and non-aged conditions. The second phenotype, referred to as the dystrophic phenotype, emerges during normal aging as homeostatic markers gradually diminish, leading to a decline in various microglial functions such as proliferation, phagocytosis, ramification, and cytokine secretion. The third phenotype, known as the disease-associated phenotype, is linked to neurodegeneration and presents an exacerbated form of the dystrophic phenotype, reflecting the diverse roles microglia can assume in different contexts.

There are three distinct phenotypes of microglia (Figure 5; Clayton et al., 2017). Under normal and non-aging circumstances, microglia exhibit a homeostatic phenotype within the adult brain. However, with advancing age, the expression of homeostasis markers within microglia gradually decreases, resulting in diminished functionality, including reduced proliferation, phagocytosis, branching, and cytokine secretion. This, in turn, can lead to the emergence of microglia with a dystrophic phenotype (von Bernhardi et al., 2015; Clayton et al., 2017). In neurodegenerative conditions, a specific subset of microglia known as DAM are observed to be present, which display altered gene expression profiles that are associated with increased inflammatory responses and reduced expression of genes involved in maintaining microglia homeostasis (Keren-Shaul et al., 2017; Krasemann et al., 2017).

Although the M1/M2 classification of microglia has recently been controversial, it may help explain the pathological relationship between inflammation and degenerative CNS diseases (Song and Suk, 2017; Guo et al., 2022). Therefore, the polarization of microglia from M1 to M2 phenotype is still used to discuss the role of microglia in AD. In the M1 state, microglia release pro-inflammatory cytokines and chemokines such as TNF-α, IL-6, IL-1β, IL-12, and CCL2. They also express iNOS, which converts arginine to NO. The accumulation of NO increases the neurotoxic effects of glutamate and damages neurons (Colonna and Butovsky, 2017). In contrast, microglia in the M2 state exhibit an anti-inflammatory phenotype, which is characterized by the production of cytokines such as IL-4, IL-10, IL-13, and TGF-β. Moreover, M2 microglia express arginase 1 to convert arginine to polyamines, which can modulate inflammation and promote tissue repair. Additionally, M2 microglia release growth factors that facilitate phagocytosis and support tissue regeneration (Zhu et al., 2019). In a state of moderate activation, microglia exhibits a dynamic balance between M1 and M2 phenotypes, enabling them to respond promptly to injurious stimuli. However, when the brain tissue is excessively stimulated, microglia undergoes a conversion toward an M1 phenotype, which is responsible for regulating inflammatory factors and causing neuronal damage. The injured neurons release toxic contents, such as Aβ, which subsequently induce microglia to adopt an M1 phenotype and release neuroimmune inflammatory factors again. This instigates a vicious cycle between damaged neurons and M1 phenotypic microglia, resulting in excessive neuroimmune inflammation and consequent neuronal death, eventually leading to brain tissue atrophy (Saito and Saido, 2018).

### 5.2. Inflammation mediated by microglia in AD

Inappropriate activation of immunity and inflammation is considered to be a significant contributor to the development of AD. Increased expression of markers associated with innate and adaptive immune system responses are observed in AD mouse models and patients’ brains (Zotova et al., 2013). GWS have identified risk loci that are strongly associated with the pathogenesis of AD, many of which are located near or within genes that are predominantly expressed in microglia (McQuade and Blurton-Jones, 2019). Furthermore, chronic neuroinflammation and glial cell activation have been shown to accompany AD pathology and partially mediate Aβ plaques and neurofibrillary tangles (NFTs). Risk factors for AD include pro-inflammatory gene polymorphisms, such as CCL3/MIP-1α and IL-6, which are produced by activated microglia (Zhao et al., 2018). Early microglia activation is associated with an increase in Aβ load and tau protein accumulation. However, when the Aβ load reaches a plateau, microglia activity subsequently decreases (Fan et al., 2017; Ismail et al., 2020).

In the early stages of AD, TREM2-dependent microglia activity slows down Aβ plaque formation (Parhizkar et al., 2019). Lots of activated microglia are clustered around the neuroinflammatory plaques in the brains of AD patients (Saito and Saido, 2018). M2 phenotype microglia are predominantly activated and play a role in inhibiting Aβ deposition and NFTs aggregation (Kulkarni et al., 2022). As neuritic plaques and NFTs increase, Aβ and p-tau deposits activate microglia receptors and trigger neuroimmune inflammatory responses, which exacerbate degeneration and neuronal death, leading to cognitive impairment and dementia (Maccioni et al., 2018). The immunogenicity of LPS and its highly pro-inflammatory effect on human neurons have been well established. In fact, elevated levels of LPS have been detected in the neocortex and hippocampus of brains affected by AD (Zhao et al., 2017a,b). Continuous aggregation of LPS-induced neuroimmune inflammation can lead to tau hyperphosphorylation and NFT formation, possibly through an increase in γ*-secretase* that results in a large accumulation of Aβ (Anthony et al., 2006). Activation of transmembrane protein receptors expressed in microglia further induces classical microglia-mediated innate immune and inflammatory responses in response to LPS (Zhao et al., 2017a,b). Finally, oxidative stress is closely related to the pathogenesis of AD (Di Bona et al., 2010). Damaging reactive oxygen species (ROS) levels are significantly higher in the AD brain than in healthy brains (Di Bona et al., 2010; Lane et al., 2018). Oxidative stress plays a significant role in the pathogenesis of AD by promoting microglia activation and morphological changes (Peters et al., 2018).

Various studies have demonstrated that microglia express different receptors, including CD36, TLR2, and TLR4, that interact with Aβ and trigger pro-inflammatory effects, resulting in chronic inflammation (Heneka et al., 2015; Su et al., 2016). In addition, microglia can activate inflammasome-related signaling upon Aβ binding, resulting in the activation of IL-1β and IL-18 through the NLR family pyrin domain containing 3 (NLRP3)/apoptosis-associated speck-like protein containing a CARD (ASC)/Caspase1 pathway, thereby contributing to the pathological progression of AD *via* detrimental inflammatory responses (Yang Y. et al., 2019; Zhao et al., 2020b). The β-Nicotinamide adenine dinucleotide phosphate oxidase (NOX) enzyme family, which produces ROS, is known to play a critical role in AD. Superoxide production, which is responsible for neuronal damage and initiates neuroinflammation-mediated progressive neuron degeneration, is attributed to NOX2. Pathological conditions lead to inflammation, which further activates NOX2 in microglia, eventually causing neuronal death (Siegel et al., 2019). Activation of NOX2 can lead to the production of superoxide, which in turn can activate NF-κB *via* various signal transduction pathways. This activation can result in the production of a large number of inflammatory factors, which may contribute to the observed neuronal death in AD (Haslund-Vinding et al., 2017; Sun et al., 2021). In summary, future therapeutic approaches for AD should also focus on reducing neuroinflammation and oxidative stress, targeting microglia activation and regulating the immune response.

## 6. Microglia and neuronal function

### 6.1. Neuronal activity

Microglia play critical roles in maintaining the integrity and plasticity of neuronal circuits in the brain. They regulate synaptic pruning and plasticity by identifying and eliminating less active synapses, preserving the strong ones (Tremblay et al., 2010; Pan and Monje, 2020). This activity is dependent on the communication between neurons and microglia. Resting microglia express the purinergic receptor, P2RY12, which enables them to respond to adenosine triphosphate (ATP) released by activated neurons. ATP functions as a chemoattractant for microglial processes, promoting communication between neurons and microglia (Peng et al., 2019; Badimon et al., 2020). Microglia break down ATP using CD39 and CD73 enzymes to produce adenosine (ADO), which then acts on the adenosine receptor, A1R, located on active neurons, thereby preventing neuronal hyperactivation (Badimon et al., 2020). This ATP-AMP-ADO-A1R-dependent feedback mechanism plays a critical role in regulating neuronal activity by preventing excessive hyperactivity that can lead to neuronal death and synaptic loss, reducing plasticity (Lepeta et al., 2016; Badimon et al., 2020). Furthermore, microglia-mediated suppression can reduce seizure severity by detecting excessive neuron excitability and decreasing neuronal firing rates.

Microglia’s frequent contact with synapses can also promote local network synchronization, followed by an increase in neuronal activity (Akiyoshi et al., 2018). Neuronal synapse activity increases when in contact with microglia, which suggests that microglia regulate neuronal activity and synchronization. *In vivo* two-photon Ca^2+^ imaging demonstrates that the synchronization of L2/3 neurons located in close proximity to one another is reduced when the normal function of microglia is disrupted, indicating that microglia can regulate neuronal activity and synchronization (Akiyoshi et al., 2018). In summary, microglia-mediated negative feedback mechanisms contribute to the homeostasis of neuronal activity, maintaining synaptic integrity and plasticity by modulating neuronal activity. Microglia play critical roles in preventing neuron hyperactivation *via* a negative feedback mechanism, reducing seizure severity, and promoting local network synchronization, providing a plausible explanation for how alterations in immune states may change synaptic plasticity.

### 6.2. Synaptic plasticity

Microglia plays a vital role in regulating synaptic plasticity. These cells constantly undergo structural changes and remodel neural circuits, contributing to the stability of LTP- the cellular mechanism of learning and memory (Poo et al., 2016; Lisman et al., 2018). The synaptic tagging and capture (STC) hypothesis suggests that weak stimulation can lead to the formation of short-lasting “synaptic tags” on certain synapses, which can be transformed into long-lasting LTP by subsequent strong stimulation from another pathway within a specific period (Frey and Morris, 1998; Nomoto and Inokuchi, 2018; Okuda et al., 2021). Under normal conditions, resting microglia promote long-term plasticity and STC. During the early phase of LTP induction, microglia set the synaptic tags that promote the stability of LTP. Eliminating microglia during this phase prevents the expression of late LTP, indicating the critical role of microglia in early STC and LTP induction (Raghuraman et al., 2019). However, once LTP is established, microglia are no longer necessary for maintaining plasticity and STC. Microglia also play a role in long-term depression (LTD), which involves the weakening of synaptic transmission. Fractalkine signaling between microglia and neurons may be involved in LTD induction. Reduced levels of fractalkine in a mouse model of Huntington’s disease result in striatal synaptic impairment and deficits in LTD induction (Kim et al., 2020). Inhibition of microglial activation prevents LTD induction, but administration of fractalkine can restore LTD (Kim et al., 2020).

The balance between proinflammatory and anti-inflammatory markers released by microglia is crucial for proper synaptic plasticity in the healthy brain (Golia et al., 2019). Disruption of this balance can lead to altered expression of inflammatory markers, resulting in decreased brain-derived neurotrophic factor (BDNF) levels and impaired phosphorylation of the AMPA receptor subunit GluR1 (Golia et al., 2019; Lin et al., 2019). The effects of proinflammatory proteins released by microglia on LTP induction appear to be transient, and the specific molecule released by microglia determines whether LTP is strengthened or impaired. For example, while overexpression of TNF-α leads to a brief increase in LTP following electrical stimulation, spatial memory deficits have been observed in TNF-α-Tg rats (Pettigrew et al., 2016). In contrast, IL-1β impairs NMDA receptor-dependent LTP at Schaffer collateral-CA1 synapses in the hippocampus (Hoshino et al., 2017). Microglia plays an essential role in regulating synaptic plasticity through their ability to release proinflammatory and anti-inflammatory mediators. The CD200/CD200R pathway and PI3K/BDNF signaling are two pathways that can modulate microglial activity and impact synaptic plasticity (Chen et al., 2017; Burk et al., 2018; Rauti et al., 2020; Saw et al., 2020). The CD200/CD200R pathway inhibits microglial activation and the release of proinflammatory mediators, promoting dendritic density and enhancing synaptic plasticity. Microglial PI3K/BDNF signaling contributes to synaptic plasticity by regulating BDNF expression, which promotes axonal branching, dendritic growth, and synapse refinement (Jung et al., 2017; Li et al., 2020; Sun et al., 2020). The balance between these molecules, including PI3K, BDNF, CREB, CD200, TNF-α, and IL-1β, determines the promotion or inhibition of synaptic plasticity. In summary, microglia plays a critical role in regulating synaptic plasticity through their ability to release proinflammatory and anti-inflammatory molecules. Resting microglia promote early STC and LTP induction, while their CD200/CD200R pathway and PI3K/BDNF signaling modulate microglial activity and impact synaptic plasticity. The specific molecule released by microglia determines whether LTP is strengthened or impaired, and the balance between proinflammatory and anti-inflammatory markers is crucial for proper synaptic plasticity in the healthy brain.

### 6.3. Aβ-dependent synapse loss

**FIGURE 6:**
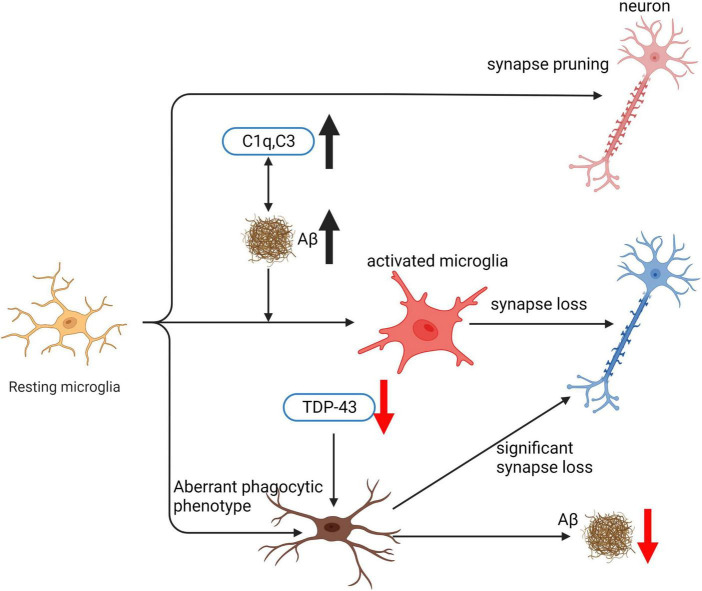
Mechanisms of synaptic loss induced by microglia under normal and pathological conditions. This illustration depicts the various pathways through which microglia contribute to neuronal synaptic loss. Under normal circumstances, microglia participate in routine synapse pruning. However, upon aggregation of Aβ, levels of complement proteins C1q and C3 rise, which in turn promote further Aβ aggregation. This initiates a vicious cycle leading to the activation of microglia, resulting in synaptic loss in neurons. Moreover, the knockout of TDP-43 causes an aberrant phagocytic phenotype in neurons, enhancing their phagocytic function. While this increase in phagocytic activity promotes Aβ clearance, it also triggers a substantial loss of synapses in the neurons.

In AD mouse models, C1q and C3 are highly upregulated, potentially exacerbating Aβ deposition and leading to synapse loss (Naito et al., 2012; Stephan et al., 2013). Studies have shown that the depletion of C1q in AD mouse models leads to reduced microglia activation and synapse loss (Fonseca et al., 2004b; Hong et al., 2016). Similarly, in a mouse model of AD lacking C3, there was reduced synapse loss and improved cognition despite an increased Aβ load (Shi et al., 2017). This suggests that complement-mediated mechanisms play a crucial role in synapse loss and cognitive impairment in AD. One possible mechanism for synapse loss in AD involves the oligomeric form of Aβ. Aβ oligomers increase the expression of the complement protein C3 in microglia, which marks synapses and promotes microglial recruitment, leading to synapse elimination (Shi et al., 2017). Injection of Aβ oligomers in mice results in upregulation of C3 levels and microglial removal of synaptic connections (Hong et al., 2016). C3 deficiency reduces phagocytic activity and protects synapses from removal, but the involvement of other complement pathway-associated molecules in AD suggests the entire complement cascade is implicated (Czirr et al., 2017; Shi et al., 2017; Zorzetto et al., 2017). The uptake of fibrillar Aβ is mediated through the C3 receptor (CD11b) and the receptor for C5a, which is upregulated around plaques and correlates with Aβ and neurofibrillary pathology (Ager et al., 2010; Fonseca et al., 2013). Inhibiting the C5aR pathway reduces Aβ pathology, neurofibrillary tangles, and improves behavior in AD mouse models (Fonseca et al., 2009). Aβ oligomers also selectively target dendritic spines, as evidenced by their colocalization with PSD-95, a marker of these structures (Lacor et al., 2004). Mechanistically, Aβ binds to glutamatergic receptors at the postsynaptic site leading to their inactivation and could act as an extracellular hook to recruit microglia to the synapse, inducing removal of the Aβ-tagged synapse (Decker et al., 2010; Li et al., 2011). One could also envision that fibrillar Aβ first recruits complement molecules, which in turn promote microglial recruitment (Eikelenboom and Veerhuis, 1996; Stoltzner et al., 2000). Because C3 is a known mediator for microglial phagocytosis of fibrillar Aβ, Aβ oligomer deposition at the synapse could induce not only microglial recruitment but also, *via* upregulation of C3, promote engulfment of synaptic structures (Maier et al., 2008; Fu et al., 2012; Figure 6).

### 6.4. Aβ-independent synapse loss

During early development, microglia play a critical role in synapse pruning (Paolicelli et al., 2011; Schafer et al., 2012), suggests that microglia’s physiological role in synapse pruning occurs well before the onset of aging or Aβ deposition. Therefore, it is plausible that the reactivation of this function during aging may not solely depend on Aβ. Selectively depleting *TDP-43* in a microglial cell line led to an aberrant phagocytic phenotype, which resulted in enhanced Aβ clearance (Paolicelli et al., 2017). Notably, mice expressing mutant APP and lacking microglial *TDP-43* displayed enhanced Aβ clearance, but at the same time, they exhibited significant synapse loss. Remarkably, the deposition of Aβ is not a prerequisite for this synaptic loss, as mice lacking any human mutant APP also exhibited a similar loss of synapses (Paolicelli et al., 2017). Another study demonstrated that 16-month-old AD mice deficient in complement C3 exhibited less synaptic loss and performed better in behavioral tests of memory, despite an increase in Aβ load (Shi et al., 2017). These findings suggest that the trajectory of synapse loss may not necessarily follow that of Aβ buildup. Taken together, these studies provide compelling evidence that microglial dysfunction, independent of Aβ pathology, may contribute to the loss of synapses. Such findings may have significant implications for the development of therapeutic strategies aimed at preventing or delaying synaptic loss in neurodegenerative diseases (Figure 6).

### 6.5. Learning and memory

Microglia have been extensively studied for their role in learning and memory. Previous research suggested a negative impact of microglia on these processes. However, recent studies have provided insights into the specific mechanisms through which microglia enhance memory by regulating synaptic pruning and neuronal remodeling. This suggests that microglia play a positive role in memory formation and consolidation (Mohammadi et al., 2020; Worthen et al., 2020; Liao et al., 2021). Microglia play a vital role in regulating the quality of memory by removing the ECM, which is involved in neuronal plasticity, learning, and memory (Vainchtein et al., 2018). This process is facilitated by the signaling of a cytokine called IL-33, which activates microglia to promote the formation of new spines and regulate neuronal plasticity in response to experiences. In mice, a loss of IL-33 receptors on microglia or neuronal IL-33 expression results in impaired synaptic plasticity and reduced precision of fear memory (Nguyen et al., 2020). Moreover, studies using transgenic mice have demonstrated the importance of IL-33 signaling in regulating memory quality. For example, IL-33 knockout mice exhibited impaired fear memory precision and reduced newborn neuron integration (Nguyen et al., 2020). Acute microglia depletion has also been found to prevent forgetting induced by pro-neurogenic drugs, highlighting the role of microglia in regulating forgetting of remote memories (Wang C. et al., 2020). Interestingly, treatment with IL-33 has been shown to improve cognitive deficits and synaptic plasticity in AD mice (Fu A. K. Y. et al., 2016). IL-33 has been found to enhance the recruitment of microglia and their interaction with plaques, resulting in increased phagocytic activity and a reduction in the size of plaques. Moreover, IL-33 administration has been shown to mitigate inflammation and upregulate anti-inflammatory genes in the brain. Additionally, microglia are involved in the process of forgetting by actively participating in adult neurogenesis through phagocytosis (Bennett and Molofsky, 2019; Wang C. et al., 2020). They identify and remove most of the newborn neurons that undergo apoptosis after formation (Diaz-Aparicio et al., 2020). When the phagocytic function of microglia is impaired for an extended period, it results in a decrease in adult hippocampal neurogenesis. Conversely, when there is a short-term deficiency in phagocytic function, it transiently enhances neurogenesis in the same area (Diaz-Aparicio et al., 2020). The morphology and function of microglia play critical roles in the regulation of learning and memory. Studies have shown that acute ablation of microglia does not impact short-term memory; however, repopulated microglia with an amoeboid morphology have been associated with enhanced memory and increased numbers of mature neurons (Gipson and Olive, 2017; De Luca et al., 2020). In summary, the studies on microglia and their impact on learning and memory underscore their intricate and fluid involvement in these processes. Microglia perform diverse functions, such as controlling memory quality through the IL-33 signaling pathway, regulating forgetting *via* phagocytosis and complement pathways, and influencing memory-related processes through their morphology and activity. These findings establish the indispensable role of microglia in learning and memory.

## 7. Therapeutic approaches

### 7.1. Therapeutic approaches based on inhibiting microglia function

#### 7.1.1. Microglial depletion

Multiple research studies have underscored the significance of specific subsets of microglia, particularly disease-associated microglia (DAM), in the progression of AD. The conjecture underpinning microglial depletion is that certain activated microglia, including DAM, may accentuate AD pathogenesis through a series of actions, such as facilitating the accumulation of Aβ plaques, amplifying neuroinflammation, and potentially influencing tau pathology (Keren-Shaul et al., 2017; Krasemann et al., 2017). DAMs, characterized by a unique transcriptional profile and often found in close proximity to amyloid plaques, are thought to have substantial impacts on neuronal health and function (Mathys et al., 2017; Deczkowska et al., 2018). Consequently, it has been proposed that therapeutic strategies should be more disease-specific and aim at selectively targeting these DAMs (Keren-Shaul et al., 2017). The implementation of microglial depletion has been achieved through various strategies, most notably using pharmacological and genetic techniques. A common method involves the use of brain-penetrating inhibitors of CSF1R, a crucial cell surface receptor for microglial survival and proliferation. In AD mouse models, these inhibitors have demonstrated improvements in cognition and reductions in both neuroinflammation and neuritic plaque formation (Dagher et al., 2015; Olmos-Alonso et al., 2016; Spangenberg et al., 2016; Sosna et al., 2018). However, the relationship between microglial depletion and amyloid pathology has been inconsistent, highlighting the need for further investigation (Casali et al., 2020). The potential of microglial depletion as a therapeutic approach must be carefully evaluated, considering the possible unforeseen consequences and the critical role microglia play in maintaining brain health (Guo et al., 2019; Oosterhof et al., 2019). Therefore, more research is warranted to further elucidate the complex roles of microglia, particularly DAM, in AD pathogenesis and treatment.

#### 7.1.2. Inhibiting the inflammatory response in microglia

Focusing on particular pro-inflammatory pathways, rather than depleting microglia, may provide a more efficacious therapeutic strategy for AD. One such pathway is the inflammasome, which plays a crucial role in microglial activation and induces the release of pro-inflammatory cytokines, ultimately leading to neuronal damage. Targeting the inflammasome pathway represents an encouraging potential treatment strategy for AD (Heneka et al., 2018; Scheiblich et al., 2020). Several medications have been developed to target different elements of the inflammasome, including purinergic receptors, caspase-1, and NLRP3 (Thawkar and Kaur, 2019). For example, purinergic receptor antagonists can regulate extracellular ATP levels, which participate in the activation of the NLRP3 inflammasome and the subsequent release of pro-inflammatory cytokines. Caspase-1 inhibitors obstruct the activation of IL-1β and IL-18, two pivotal cytokines in the inflammasome pathway, thereby attenuating the inflammatory response. Direct inhibitors of NLRP3 can hinder its activation, oligomerization, or interaction with other inflammasome components, ultimately preventing inflammasome assembly and downstream inflammatory signaling. These therapeutic interventions hold the potential to reduce neuroinflammation, alleviate neuronal damage, and decelerate AD progression. Nevertheless, it is vital to carry out additional pre-clinical and clinical research to determine the safety and efficacy of these drugs, as well as to comprehend the potential side effects and long-term consequences of targeting specific inflammasome components. By deepening our understanding of the molecular mechanisms and interactions within the inflammasome pathway, we can continue to develop innovative and targeted therapies for AD that minimize adverse effects and maximize therapeutic potential.

##### 7.1.2.1. Targeting purinergic receptors to inhibit microglial inflammation in AD

Emerging as potential therapeutic targets for AD, purinergic receptors, especially the P2 × 7R subtype found on microglia, play dual roles in neuroprotection and neurodegeneration (Burnstock, 2016; Woods et al., 2016). P2 × 7R antagonism may offer significant therapeutic benefits for addressing neuroinflammation and other neurodegenerative conditions, as it occurs exclusively under high ATP concentrations, which are believed to increase during the progression of neuroinflammatory diseases (Sperlágh and Illes, 2014; Bhattacharya, 2018; Burnstock and Knight, 2018). Research has highlighted the importance of P2 × 7R in the development of neurodegenerative disorders through both *in vitro* and *in vivo* studies. In the context of AD, characterized by reactive microgl lia and Aβ plaques, Aβ is known to stimulate neuroinflammation *via* P2 × 7R modulation (Sanz et al., 2009). Targeting P2Y2R during the early stages of AD could be beneficial due to its role in tissue repair (Peterson et al., 2010). Brilliant blue G (BBG), a P2 × 7R antagonist derived from the blue food dye FD&C blue No. 1, has shown promise in treating neurological disorders due to its unique ability to cross the blood-brain barrier. Demonstrating a favorable safety profile in healthy animal models, BBG has potential clinical applications (Borzelleca et al., 1991). Widely recognized for its effectiveness on P2 × 7R in various species, BBG has been observed to enhance cognition and promote dendritic spine development in Aβ_42_-injected rats (Chen et al., 2014). Furthermore, BBG exhibits neuroprotective properties by counteracting the inflammatory responses induced by P2 × 7R agonists in Aβ-peptide-injected rat brains (Ryu and McLarnon, 2008). GlaxoSmithKline has developed a P2 × 7 receptor antagonist, GSK1482160, which exhibits superior CNS penetration and the ability to be radiolabeled with 11C. The radio labeled 11C-GSK1482160 displays high P2 × 7 selectivity and functions as a neuroinflammation biomarker in LPS-challenged rats (Territo et al., 2017).

##### 7.1.2.2. Inhibiting NLRP3 inflammasome activation

Several compounds have demonstrated potential in modulating the NLRP3/caspase-1 inflammasome pathway in AD. Pterostilbene, for instance, has been found to be effective in reducing inflammasome activation and lowering the secretion of proinflammatory mediators in BV-2 microglia at a concentration of 10 μM (Li J.-J. et al., 2018; Li Q. et al., 2018). Similarly, JC-124, a small molecule NLRP3 inflammasome inhibitor, led to reduced levels of Aβ deposition and decreased levels of soluble and insoluble Aβ_1–42_ in the brain of TgCRND8 mice (Yin et al., 2018). MCC950, a NLRP3 inhibitor, has been found to prohibit inflammasome activation and IL-1β release from microglia. Moreover, it promoted Aβ phagocytosis in the APP/PS1 mouse model of AD (Dempsey et al., 2017). In addition, Benzyl isothiocyanate (BITC), a naturally occurring compound found in cruciferous vegetables, has been found to inhibit the secretion of IL-1β, inflammasome activation, and proinflammatory mediators in BV2 microglial cells (Lee et al., 2016). Furthermore, Stavudine, a nucleoside reverse transcriptase inhibitor, has been found to downregulate NLRP3 inflammasome activation and Aβ autophagy in cell cultures of THP-1-derived macrophages induced by Aβ (La Rosa et al., 2019). Similarly, Artemisinin, obtained from Artemisia annua and well-known for its antimalarial effect, has been found effective in treating AD by inhibiting activation of NF-κB and NLRP3 inflammasome in 5-month-old *APPswe/PS1dE9* transgenic mice (Shi et al., 2013). Finally, Dihydromyricetin (DHM) has been found to decrease activation of the NLRP3 inflammasome and reduce memory and cognition deficits in APP/PS1 double-transgenic mice (Feng et al., 2018). These findings suggest that targeting the NLRP3 inflammasome may offer a promising therapeutic strategy for the treatment of AD.

##### 7.1.2.3. Caspase-1 inhibition: a potential strategy for modulating microglial activation in AD

Caspase 1 has gained attention as a potential therapeutic target for addressing age-related cognitive decline and AD. Bacopa, a naturally occurring compound, exhibits inhibitory effects on several enzymes involved in neuroinflammation and neurodegeneration, including Matrix Metalloproteinase-3 (MMP-3), caspase 1, and caspase 3. In a cell-free assay, Nemetchek et al. (2017) showed that Bacopa effectively suppressed the activity of these enzymes, supporting its potential as a therapeutic agent for AD. Additionally, VX-765, a selective caspase-1 inhibitor, has demonstrated promising effects in AD mouse models. It has been found to attenuate Aβ peptide accumulation, mitigate brain inflammation, and preserve normal synaptophysin protein levels in the mouse hippocampus. These outcomes were dose-dependent and observed in J20 mice (Flores et al., 2018). Other studies have also reported the neuroprotective effects of caspase-1 inhibition, further emphasizing its potential as a therapeutic strategy for AD (Kaushal et al., 2015).

##### 7.1.2.4. RIPK1 inhibition: a pathway to mitigate microglial-driven pathology in AD

The role of receptor-interacting serine/threonine kinase 1 (RIPK1) activation in microglia is thought to be crucial in the development of AD. While RIPK1 has been linked to the inflammasome response, recent research indicates that its function in this process is primarily independent of its kinase activity (Malireddi et al., 2020). Notably, increased RIPK1 kinase activity has been observed in animal models of AD, amyotrophic lateral sclerosis (ALS), and multiple sclerosis (MS), as well as in human patient samples, implying a potential contribution of this protein to the pathogenesis of these neurodegenerative disorders (Yuan et al., 2019). Various pre-clinical studies have underscored the potential protective effects of utilizing either a kinase-dead mutant of RIPK1 (RIPK1D138N) or pharmacological RIPK1 inhibitors in diverse neurodegenerative disease models. RIPK1 activity is known to modulate cell death processes such as apoptosis and necroptosis and to regulate the production of inflammatory cytokines in specific cell types, including microglia (Degterev et al., 2019; Yuan et al., 2019). Intriguingly, RIPK1 activity has also been demonstrated to regulate metabolic adaptation and reactive oxygen species production in response to TNF-a, which may further contribute to pathological microglial cell states (Zhang et al., 2009). Owing to these findings, ongoing clinical trials are exploring the use of small-molecule RIPK1 inhibitors for AD. Collectively, these studies suggest that targeting RIPK1 may offer therapeutic potential in the management of neurodegenerative diseases.

### 7.2. Therapeutic approaches based on enhancing microglia function

#### 7.2.1. Exploring TREM2: modulating microglial activity as a therapeutic strategy for AD

**FIGURE 7:**
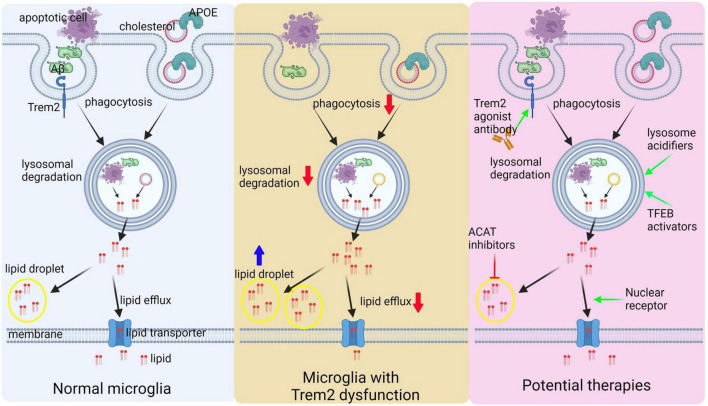
Outlines therapeutic strategies targeting microglial lipid and lysosomal functions. Phagocytic activity and apolipoprotein uptake, such as *APOE*, are crucial for delivering lipids to endolysosomal compartments for degradation. During this process, cholesterol is liberated from digested phagocytic cargo, such as apoptotic cells. Subsequently, cholesterol can exit the endolysosomal compartment and be esterified into smaller lipid molecules. These lipids can either be stored in lipid droplets or exported from the cell *via* lipid transporters. *TREM2* dysfunction can result in various microglial defects, including diminished phagocytosis, impaired lysosomal degradation, and compromised cholesterol efflux, as well as the accumulation of neutral lipids in lipid droplets. Multiple therapeutic opportunities exist for targeting microglial function in AD. *TREM2*-targeting antibodies can enhance phagocytosis and lysosomal function. Small molecules targeting ACAT can reduce lipid droplet formation, while various nuclear receptors can promote cholesterol efflux. Additionally, lysosomal function activation can be achieved through small molecules that induce lysosomal acidification or by stimulating the activation of *TFEB* family members to enhance lysosomal function.

Although TREM2-dependent activities may not significantly reduce amyloid plaque load in mouse models, they can promote plaque compaction and alleviate plaque-associated neuritic pathology (Ulrich et al., 2014; Wang et al., 2016; Meilandt et al., 2020). As a result, TREM2 has emerged as a primary therapeutic target for AD, with researchers exploring methods for selective modulation to achieve favorable outcomes. Disease-associated *TREM2* variants can result in loss of function in mouse and cellular models, implying that enhancing TREM2 function could be beneficial (Heslegrave et al., 2016; Piccio et al., 2016; Suárez-Calvet et al., 2019). Indirect evidence from AD patients suggests that higher TREM2 levels may have protective effects on disease progression. Several independent cohorts have reported increased levels of soluble TREM2 (sTREM2) concentrations in cerebrospinal fluid (CSF) early in the disease, which could serve as a potential marker for full-length TREM2-mediated signaling. Elevated sTREM2 levels have been detected in patients with mild cognitive impairment and dominant inherited AD, up to 5 years before the estimated year of onset (Suárez-Calvet et al., 2016a). However, no correlation was found between sTREM2 and amyloid deposition, suggesting that TREM2 expression might increase in response to tau-related cellular pathology. Research has also found that sTREM2 levels rise during physiological aging in humans and mice, with TREM2-dependent increases in microglial nodules observed during aging in mice (Suárez-Calvet et al., 2016a; Kleinberger et al., 2017). It is hypothesized that these microglial accumulations are a response to myelin damage, supported by studies in demyelination models (Poliani et al., 2015; Nugent et al., 2020). Additionally, in humans, a higher ratio of CSF sTREM2 over phospho-tau predicted slower clinical progression and was associated with slower cognitive decline (Ewers et al., 2019). The cumulative evidence suggests that TREM2 plays a beneficial role in the CNS, which could be amplified to prevent or slow the progression of sporadic AD. Agonist antibodies binding to the extracellular region and initiating receptor signaling may hold potential for AD treatment (Figure 7; Cignarella et al., 2020; Price et al., 2020; Schlepckow et al., 2020; Wang S. et al., 2020). Modulating TREM2 activity represents a promising avenue for developing effective therapeutic interventions for AD. Mouse TREM2 studies have shown that antibody stimulation enhances microglial chemotaxis, clustering around Aβ plaques, and phagocytosis of cellular debris, possibly facilitating Aβ plaque removal (Wang et al., 2016; Mazaheri et al., 2017; Parhizkar et al., 2019). However, research on an anti-mouse TREM2 antibody that competes for ligand binding and inhibits cell autonomous signaling revealed that TREM2 antagonism significantly worsened the outcome in experimental autoimmune encephalomyelitis (EAE). Currently, three promising strategies for modulating TREM2 activity have emerged. Two companies have identified antibodies that enhance TREM2 signaling through crosslinking (Cheng et al., 2018; Cignarella et al., 2020; Price et al., 2020; Wang S. et al., 2020), while the third approach aims to prevent shedding of the extracellular domain by ADAM family proteases (Wunderlich et al., 2013; Schlepckow et al., 2020). However, selectively inhibiting TREM2 shedding is preferred over inhibiting ADAM10, which cleaves other vital physiological substrates in the brain (Kuhn et al., 2016; Schlepckow et al., 2020). One promising antibody clone, 4D9, binds to an epitope only 12 amino acids N-terminal to the TREM2 protein, effectively obstructing the shedding of soluble TREM2. *In vitro* biochemical assays have demonstrated that 4D9 can impede the cleavage of the TREM2 stalk peptide, activating DAP12/phosphorylation/Syk signaling, which is crucial for downstream signaling (Cignarella et al., 2020; Price et al., 2020; Wang S. et al., 2020). Investigations have revealed that 4D9 efficiently binds sTREM2 in the CSF when administered at high doses peripherally, suggesting that it can engage the target in the CNS *in vivo* (Wang S. et al., 2020). Studies have assessed the effects of AL002a/c, a humanized monoclonal IgG1 antibody that binds to TREM2 and activates the TREM2 signaling pathway, and 4D9 on Aβ accumulation in transgenic AD mouse models (Price et al., 2020; Schlepckow et al., 2020; Wang S. et al., 2020). Surprisingly, neither AL002c nor 4D9 enhanced the clustering of microglia around amyloid plaques, while AL002a did. In contrast, AL002a significantly reduced the total Aβ plaque load in the same model and decreased fibrillar Aβ within the hippocampus. Furthermore, both AL002a/c improved behavior in 5 × FAD mice and reduced plasma neurofilament light chain, a marker for neurodegeneration (Wang S. et al., 2020). In summary, the AL002 family of antibodies shares numerous properties, but they display divergent activities toward Aβ clearance and microglial clustering. The reasons for these discrepancies remain unclear but could stem from differences in mouse models or dosing paradigms utilized in these studies.

#### 7.2.2. Enhancing lysosomal activity

Microglial phagocytosis is a multifaceted process that necessitates substantial alterations in microglial gene expression and a robust lysosomal degradative capacity. Microglia express high levels of genes controlling lysosomal function, including hydrolases and regulators such as cathepsins, lipases, prosaposin, and progranulin. However, AD may impair lysosomal degradative capacity. For example, Aβ aggregates can undermine microglial endolysosomal membrane integrity, potentially disrupting lysosomal compartment proton gradients and releasing cathepsins into the cytosol (Heneka et al., 2018). Additionally, lysosomal dysfunction is evident in aged murine microglia, characterized by lipofuscinosis (Safaiyan et al., 2016). Enhancing lysosomal function might be a viable therapeutic approach to improve microglial function (Figure 7). Targeting lysosomal pH control, such as increasing the stability or activity of ClC-7 in microglia or promoting V-type ATPase activity, may facilitate the degradation of age- and disease-associated phagocytic cargoes (Majumdar et al., 2011). An alternative strategy to boost lysosomal biogenesis involves enhancing signaling pathways controlling this process at the transcriptional level. Key regulators of genes encoding hydrolases, membrane proteins, proton pumps, and macroautophagy factors crucial for proper lysosomal function include transcription factor EB (*TFEB*) and its related family members *TFE3*, *TFEC*, and *MITF*. These transcription factors act as master regulators, playing a critical role in ensuring lysosomal functionality (Sardiello et al., 2009). In microglia, nutrient deprivation and mTORC1 signaling downregulation or lysosomal dysfunction activate the *TFEB* pathway (Sardiello et al., 2009). The coordinated lysosomal expression and regulation (CLEAR) network is activated *in vivo* in lysosomal storage disease models, including Ids and Grn KO, though specific brain cell types might be differentially affected (Götzl et al., 2018; Ullman et al., 2020). In microglia, not only nutrient deprivation but also immune stimuli such as TLR agonists can activate *TFEB* and *TFE3*. This activation leads to the induction of various pro-inflammatory cytokines, as well as lysosomal and autophagic genes. While the activation of the *TFEB*/*TFE3* pathway may promote lysosomal degradation and autophagy, it could also enhance inflammatory responses, resulting in either beneficial or detrimental outcomes depending on the disease or disease stage (Götzl et al., 2018; Ullman et al., 2020). From a therapeutic perspective, exploiting endogenous regulators of the *TFEB* pathway in microglia, such as mTORC1 pathway antagonists, may be a potential approach (Puertollano et al., 2018). Enhancing lysosomal biogenesis in microglia could help clear disease-associated lysosomal cargoes, including protein aggregates like Aβ and tau, as well as lipid-rich cellular debris.

#### 7.2.3. Enhancing lipid processing

The study of LOAD genetics has pinpointed lipid metabolism as a crucial risk factor (Efthymiou and Goate, 2017). The discovery of *APOE4* as the primary genetic susceptibility gene for AD has raised the possibility that lipid transport, including cholesterol and triglyceride metabolism, might be implicated in the disease mechanism (Corder et al., 1993). It has been demonstrated that APOE isoforms differentially impact peripheral lipid metabolism (Leduc et al., 2010; Zhao et al., 2020c), and subsequent investigations have concentrated on the relationship between *APOE* and Aβ deposition in the brain parenchyma and amyloid efflux from the brain (Huynh et al., 2017). Recent findings suggest that the lipidation degree of *APOE* may influence amyloidogenesis, as poorly lipidated *APOE4* has shown a higher propensity for both aggregation and Aβ seeding (Huynh et al., 2017). Additionally, microglial *APOE* has been found to play a critical role in immunometabolism, with dysfunction in these cells leading to two distinct types of cholesterol-processing impairments: the accumulation of lysosomal cholesterol and cholesterol crystals, as well as the buildup of cholesteryl esters, the stored form of cholesterol, within lipid droplets (Cantuti-Castelvetri et al., 2018; Nugent et al., 2020). Cholesteryl ester accumulation has been observed in microglia that lack either *TREM2* or its signaling component, phospholipase Cγ2, encoded by the LOAD-associated *PLCG2* gene (Andreone et al., 2020; Nugent et al., 2020). This observation aligns with prior lipidomic analyses of LOAD patient brain samples and 5 × FAD mouse models (Chan et al., 2012). Furthermore, studies involving *APOE* knockout microglia and *TREM2* knockout induced microglia have highlighted the indispensable role of apolipoprotein in central nervous system cholesterol transport during demyelination events. These investigations emphasize the importance of understanding lipid metabolism in the context of neuroinflammation and neurodegeneration (Cantuti-Castelvetri et al., 2018; Andreone et al., 2020; Nugent et al., 2020). The presence of lipid droplets containing cholesteryl esters and cholesterol in lysosomes leads to the “foamy” appearance of diseased microglia, which can cause lipotoxicity and cellular stress (Moore and Tabas, 2011; Olzmann and Carvalho, 2019). This can contribute to decreased cell survival, heightened pro-inflammatory signaling, oxidative stress, and inflammasome activation (Moore and Tabas, 2011). To address these issues, nuclear receptors, such as PPARs, LXRs, and RXRs, have been targeted as potential therapeutics to promote lipid efflux while reducing inflammation (Moutinho and Landreth, 2017). Although some agonists have shown efficacy in AD mouse models, such as reducing Aβ burden and exerting anti-inflammatory actions in the brain, their clinical efficacy remains uncertain (Moutinho and Landreth, 2017). For example, LXR agonists, such as GW3965, have demonstrated profound effects in microglia by rescuing cholesterol and cholesteryl ester accumulation in *APOE* KO microglia and *TREM2* KO iMG (Cantuti-Castelvetri et al., 2018; Nugent et al., 2020). Furthermore, targeting ACAT1, the enzyme that catalyzes the esterification of cholesterol, through inhibition or ablation, has demonstrated the potential to decrease Aβ and tau pathology in mouse models of AD. This effect is believed to be mediated through modulation of both APP processing and autophagy (Puglielli et al., 2001; Hutter-Paier et al., 2004; Shibuya et al., 2015). Reducing cholesteryl esters may decrease oxidized cholesteryl ester species and minimize the deleterious effects of lipid peroxidation (Nugent et al., 2020). However, reducing cholesterol esterification may increase cellular levels of free cholesterol, which requires efficient efflux mechanisms for its elimination from the cells. In conclusion, understanding the critical role of lipid metabolism in LOAD and identifying potential therapeutic targets may lead to more effective treatments for this debilitating disease (Figure 7).

## 8. Summary and outlook

In this review, we have provided a comprehensive overview of the critical role microglia play in AD, covering key aspects such as the associated genetic mutations, microglial phenotypes, phagocytic functions related to Aβ and apoptotic cells, the involvement of inflammatory pathways, and the relationship between microglia, synaptic loss, and synaptic plasticity. Moreover, we have highlighted the development of AD-related therapeutics targeting microglia and the current limitations in our understanding of microglial biology.

In conclusion, it is evident that microglia are crucial players in the pathogenesis of AD, and a deeper understanding of their diverse functions and interactions with other cellular components in the brain will be vital for the development of effective therapeutic strategies. However, despite considerable progress in recent years, there remain significant gaps in our knowledge of microglial biology, particularly concerning their heterogeneity, precise mechanisms of action, and the interplay between various signaling pathways.

Future research should continue to unravel the complexity of microglial biology, explore the underlying molecular mechanisms that drive their diverse functions, and investigate the potential of modulating microglial activity to halt or reverse disease progression. Additionally, the development of novel *in vitro* and *in vivo* models that more accurately recapitulate the human disease will be crucial for the validation of new therapeutic targets and for testing candidate drugs. Advances in single-cell sequencing technologies and systems biology approaches will undoubtedly contribute to a better understanding of microglial heterogeneity and their functional *STAT*es in AD.

One notable limitation of current research is the lack of standardized criteria for defining microglial phenotypes and functional states, which may lead to inconsistencies in the literature and hinder the development of targeted therapies. Furthermore, many studies focus on individual aspects of microglial biology, which may not fully capture the complex interplay between various microglial functions in the context of AD.

The design and implementation of clinical trials that specifically target microglial pathways should consider factors such as disease stage, genetic background, and individual patient characteristics to achieve optimal therapeutic outcomes. Moreover, addressing the current limitations in our understanding of microglial biology and function will be essential to harness the potential of microglia-targeted therapies for the prevention, management, and eventual cure of AD. Together, these efforts will pave the way for a new era of precision medicine, capitalizing on the promise of microglia-targeted interventions for AD.

## Author contributions

JM conceptualized the study, performed the literature search, and drafted the manuscript. YY, HM, and YL were responsible for critically reviewing and analyzing the literature. JZ, CL, and MY contributed to data interpretation and provided expert input. JL supervised the overall project, provided critical feedback, and revised the manuscript for important intellectual content. All authors contributed to the final version of the manuscript and approved it for submission.

## Figures and Tables

**Table:** 

Abbreviation	Full name
ABCA7	ATP-binding cassette sub-family A member 7
ACT	Antichymotrypsin
AD	Alzheimer’s disease
ADAM	A disintegrin and metalloprotease
ADO	Adenosine
ADSP	AD Sequencing Project
AKAP9	A-kinase anchor protein 9
ALS	Amyotrophic lateral sclerosis
ANXA1	Annexin A1
AP-1	Activator protein 1
APP	Amyloid precursor protein
Arg-1	Arginase 1
ATP	Adenosine triphosphate
Aβ	Amyloid beta
BAI1	Brain-specific angiogenesis inhibitor 1
BBB	Blood-brain barrier
BBG	Brilliant blue G
BDNF	Brain-derived neurotrophic factor
BIN1	Bridging integrator 1
BITC	Benzyl isothiocyanate
C2CD3	C2 Domain Containing 3 Centriole Elongation Regulator
CaMKKβ	Calcium/calmodulin-dependent protein kinase kinase-β
CAR-Ps	Chimeric antigen receptor-phagocytic receptors
CD33	Siglec-3
CLEAR	Coordinated lysosomal expression and regulation
CLSTN2	Calsyntenin 2
CNS	Central Nervous System
CR1	Complement receptor 1
CTNNA2	N-catenin and Catenin alpha-2
CX3CR1	CX3C chemokine receptor 1
DAM	Disease-associated microglia
DAMPs	Danger-associated molecular patterns
EAE	Experimental autoimmune encephalomyelitis
ECM	Extracellular matrix
ERK1/2	Extracellular signal-regulated kinase 1/2
EXOC5	Exocyst complex component 5
FERMT2	Fermitin family homolog 2
FNBP1L	Formin-binding protein 1-like
GAS2L2	GAS2-like protein 2
Gas6	Growth arrest-specific protein 6
GPCRs	G-protein-coupled receptors
GULP1	PTB domain-containing engulfment adapter protein 1
GWAS	Genome-wide association studies
HO-1	Heme oxygenase-1
IκB	Inhibitors of NF-κB
IKK	IκB kinase
IL-4	Interleukin 4
IRAK	Interleukin-1 receptor-associated kinase
JAK/STAT	Janus kinase/signal transducer and activator of transcription
KIF2A	Kinesin heavy chain member 2A
LAP	LC3-associated phagocytosis
LHX9	LIM Homeobox 9
LKB1	Liver kinase B1
LOAD	Late-onset Alzheimer’s Disease
LPC	Lipocortin
LPS	Lipopolysaccharide
LRP1	LDL-receptor-associated protein 1
LTD	Long-term depression
LTP	Long-term potentiation
MAPK	Mitogen-activated protein kinases
MFG-E8	Milk fat globule-EGF factor 8 protein
MS	Multiple sclerosis
MyD88	Myeloid differentiation factor 88
NALCN	Sodium Leak Channel, Non-Selective
NDRG2	NDRG Family Member 2
NF-κB	Nuclear factor-κB
NFTs	Neurofibrillary tangles
NICD	Notch intracellular domain
NLRs	Nucleotide-binding oligomerization domain-like receptors
NO	Nitric oxide
NOX	Nicotinamide adenine dinucleotide phosphate oxidase
NSF	Vesicle-fusing ATPase
OECs	Olfactory ensheathing cells
P2 × 7R	P2 × 7 receptor
PAMP	Pathogen-associated molecular patterns
PILRA	Paired immunoglobin-like type 2 receptor alpha
PI3K	Phosphoinositide 3-kinase
PGC-1α	Peroxisome proliferator-activated receptor γ coactivator 1α
PKB	Protein kinase B
PLC	Phospholipase C
PPAR-γ	Peroxisome proliferator-activated receptor gamma
PRKCH	Protein kinase C
PRRs	Pattern recognition receptors
PSD-95	Postsynaptic density protein-95
PSEN1	Presenilin 1
PtdSer	Phosphatidylserine
RAGE	Receptor for advanced glycation end products
Rac1	Ras-related C3 botulinum toxin substrate 1
RIPK1	Receptor-interacting serine/threonine kinase 1
ROS	Reactive oxygen species
RXRs	Retinoid X receptors
S1P	Sphingosine-1-phosphate
SAP	Serum amyloid protein
SAβ	Soluble amyloid beta
SEL1L	Suppressor of lin-12-like protein 1
sFKN	Soluble fractalkine
SIGLECs	Sialic acid-binding immunoglobulin-like lectins
SIRPα	Signal-regulatory protein alpha
Sirt1	Sirtuin 1
SLC24A4	Solute carrier family 24 member 4
SNP	Single nucleotide polymorphism
SOCS	Suppressor of cytokine signaling
SORL1	Sortilin related receptor 1
SRA	Scavenger receptor A
SR-A1	Class A1 scavenger receptors
SR-MARCO	Scavenger receptor and macrophage receptor with collagenous structure
STAT	Signal Transducer and Activator of Transcription
STC	Synaptic tagging and capture
SYTL3	Synaptotagmin-like 3
TAM	Tyrosine kinases
TAK1	Transforming growth factor-β-activated kinase-1
TDP-43	43 kDa TARDNA binding protein
TFEB	Transcription factor EB
TFEC	Transcription factor EC
MITF	Microphthalmia-transcription-factor
TG2	Transglutaminase 2
TIAF1	TGFB1-induced antiapoptotic factor 1
TLR s	Toll-like receptors
TMEM132A	Transmembrane Protein 132A
TNF	Tumor necrosis factor
TRAF6	Tumor necrosis factor receptor associated factor-6
TREM2	Triggering receptor expressed on myeloid cells 2
VR	Vitronectin receptor
WES	Whole exome sequencing
WGS	Whole genome sequencing
WNT7A	Wnt Family Member 7A
ZNF655	Zinc Finger Protein 655
